# Association of the phase angle with type 2 diabetes and related traits: results from two prospective KORA studies

**DOI:** 10.1038/s41387-026-00425-x

**Published:** 2026-05-09

**Authors:** Feiling Ai, Marie-Theres Huemer, Wolfgang Rathmann, Michael Roden, Christian Herder, Tanja Zeller, Wolfgang Koenig, Jana Nano, Michael Drey, Annette Peters, Barbara Thorand

**Affiliations:** 1 Institute of Epidemiology, Helmholtz Zentrum München, German Research Center for Environmental Health (GmbH), Neuherberg, Germany; 2 Institute for Medical Information Processing, Biometry, and Epidemiology (IBE), Faculty of Medicine, LMU Munich, Pettenkofer School of Public Health, Munich, Germany; 3 Institute for Biometrics and Epidemiology, German Diabetes Center, Leibniz Center for Diabetes Research at Heinrich Heine University Düsseldorf, Düsseldorf, Germany; 4 German Center for Diabetes Research (DZD), Partner Düsseldorf, Neuherberg, Germany; 5 Department of Endocrinology and Diabetology, Medical Faculty and University Hospital Düsseldorf, Heinrich Heine University Düsseldorf, Düsseldorf, Germany; 6 Institute for Clinical Diabetology, German Diabetes Center, Leibniz Center for Diabetes Research at Heinrich Heine University Düsseldorf, Düsseldorf, Germany; 7 Institute for Cardiogenetics, University of Lübeck, University Hospital Schleswig-Holstein, University Heart Center Lübeck, Lübeck, Germany; 8 German Center for Cardiovascular Research (DZHK), Partner Site Nord, Lübeck, Germany; 9 German Heart Center, TUM University Hospital, Munich, Germany; 10 German Center for Cardiovascular Research (DZHK), Partner Site Munich Heart Alliance, Munich, Germany; 11 Institute of Epidemiology and Medical Biometry, University of Ulm, Ulm, Germany; 12 Department of Radiation Oncology, Klinikum rechts der Isar, Technical University of Munich (TUM), Munich, Germany; 13 Department of Medicine IV, LMU University Hospital, LMU Munich, Munich, Germany; 14 German Center for Diabetes Research (DZD), Partner München-Neuherberg, Neuherberg, Germany

**Keywords:** Type 2 diabetes, Epidemiology

## Abstract

**Objectives:**

To investigate associations of the bioelectrical impedance analysis (BIA)-derived phase angle (PhA), an indicator of body cell mass, hydration status, and cell membrane integrity, with type 2 diabetes (T2D), prediabetes, and glycemic and insulin-related traits.

**Methods:**

Using data from the Cooperative Health Research in the Region of Augsburg (KORA) S3/S4 studies, we analyzed 7728 participants aged 25–74 years for prevalent T2D and 7006 participants who did not have diabetes at baseline for incident T2D. A subsample aged 55–74 years at S4 was followed to assess incident oral glucose tolerance test (OGTT)-defined prediabetes or T2D (prediabetes/T2D), and glycemic and insulin-related traits (S4/F4/FF4). The PhA was calculated from BIA 2000-S at 50 kHz. Logistic and Cox regressions were applied for binary outcomes, and two-level growth models for continuous traits.

**Results:**

In S3/S4, 324 participants had prevalent T2D at baseline, and 707 developed T2D during a median 15.7-year follow-up. In S4/F4/FF4, during up to 14 years of follow-up, 251 out of 626 normoglycemic participants at S4 developed incident prediabetes/T2D, and 792–804 participants without diabetes at S4 had three repeated measurements of continuous traits. The PhA (per 1-degree) was positively associated with incident T2D (hazard ratio [HR] and 95% confidence interval [CI] in S3/S4: 1.37 [1.21–1.54]) and incident prediabetes/T2D (HR [95% CI] in S4/F4/FF4: 1.33 [1.07–1.67]) without sex differences. The PhA (per 1-degree) was also positively associated with fasting glucose (beta [95% CI]: 1.2% [0.1–2.2%]) and insulin resistance (beta [95% CI]: 7.0% [2.3–11.7%]) cross-sectionally, and with changes in 2-h glucose longitudinally (beta [95% CI]: 4.5% [2.3–6.7%]) (S4/F4/FF4). In contrast, the PhA (per 1-degree) was inversely associated with prevalent T2D (odds ratio [95% CI] in S3/S4: 0.72 [0.56–0.93]) in men only.

**Conclusions:**

The PhA at 50 kHz had stage-dependent associations with glucose metabolism, with higher values observed during subclinical stages and lower values after diabetes manifestation.

## Introduction

Bioelectrical impedance analysis (BIA) is a non-invasive and relatively low-cost method for body composition assessment and has been implemented as an alternative to more invasive and costly techniques such as dual-energy X-ray absorptiometry, computerized tomography, and magnetic resonance imaging [1]. A key BIA-derived parameter is the phase angle (PhA), which is calculated from two raw BIA measurements of capacitive reactance (Xc) and resistance (R) [2]. The PhA serves as an indicator of body cell mass (BCM), cellular integrity, and tissue hydration status, particularly extracellular and intracellular water distribution [ECW/ICW] [3], supported by its associations with protein markers related to cell proliferation [4]. A higher PhA is mainly characterized by greater fat-free mass (FFM) and lower ECW/ICW ratios for both sexes [5]; while a lower PhA has been associated with detrimental cellular changes, such as reduced BCM, increased ECW/ICW ratios, and impaired cellular integrity [5, 6]. Throughout the lifespan, PhA values increase progressively from infancy to adolescence, stabilize during adulthood, and gradually decrease from around 50 years onwards [6]. Men tend to have higher values than women across life, which may result from their greater skeletal muscle mass [SMM] [6]. The PhA is positively associated with body mass index (BMI) in individuals with normal or moderately elevated BMI; however, an inverse association has been observed when BMI exceeded 35 kg/m² [7] or 40 kg/m² [8].

Recently, the PhA has emerged as a promising biomarker for assessing inflammation, oxidative stress, muscle composition, cardiovascular risk, and nutritional status in metabolic diseases [9–11]. Few prior studies have indicated that the PhA may reflect underlying metabolic and cellular alterations in individuals with diabetes and related complications [12]. However, existing cross-sectional studies have yielded inconsistent findings and were constrained by small sample sizes and minimal adjustments for potential confounders [13–18]. Moreover, no study to date has explored the longitudinal associations of the PhA with incident type 2 diabetes (T2D), incident prediabetes, or with changes in glycemic and insulin-related traits, leaving its potential role in early glucose dysregulation unclear.

Therefore, using data from two population-based prospective cohorts, the present study aimed (1) to investigate the associations of the PhA with prevalent T2D and incident T2D; (2) to examine the longitudinal associations of the PhA with incident oral glucose tolerance test (OGTT)-defined prediabetes or T2D among normoglycemic participants; and (3) to assess the cross-sectional and longitudinal associations of the PhA with glycemic and insulin-related traits among participants without known or newly OGTT-defined diabetes at baseline.

## Materials and methods

### Research design and study participants

The Cooperative Health Research in the Region of Augsburg (KORA) cohort (Fig. S1) is a regional research platform for population-based cohort studies in Southern Germany (https://www.helmholtz-munich.de/en/epi/cohort/kora) [19]. Men and women with a broad age range (S1: 25–64 years; S2–S4: 25–74 years) were randomly selected, stratified by urban/rural region, sex, and 10-year age groups to ensure representativeness of the general population. The present study used data from two prospective KORA studies (S3 and S4) with available BIA measurements.

S3/S4 studies (Fig. S2): We used data from participants aged 25–74 years at baseline who were enrolled in S3 (1994–1995) and S4 (1999–2001). The S3 study originated from the Monitoring of Trends and Determinants in Cardiovascular Diseases (MONICA) Augsburg study. Follow-up examinations and written questionnaires were used to assess the health status of participants until 2016 [19, 20]. Participants were excluded if they had duplicate records in S3 and S4, lacked informed consent, were ineligible for BIA measurements according to the protocol (such as pregnant women, participants with severe edema, electronic/metal implants or portable electronic devices, joint prostheses, amputations, paralysis, and bandage), had cancer diagnoses or missing information on cancer status within the last year that could affect BIA measurements, had extreme PhA values, had prevalent diabetes other than T2D, or had incomplete data at baseline. The cross-sectional analyses for prevalent T2D included 7728 participants, while the longitudinal analyses for incident T2D comprised 7006 participants who did not have known diabetes at baseline.

S4/F4/FF4 studies (Fig. S3): We further used data from a subsample of 1653 participants aged 55–74 years who were initially examined at S4 and were followed up at F4 (2006–2008) and/or FF4 (2013–2014) examinations. In this subsample, all participants fasted for at least 8 h prior to their visit to the study center, and an OGTT was performed to ascertain prediabetes and previously undiagnosed T2D at each visit (S4, F4, and FF4), along with measurements of five continuous glycemic and insulin-related traits. After exclusions similar to those in S3/S4 studies, 863 participants without known or newly OGTT-defined T2D at S4 had available OGTT data for further analyses. First, for analyses regarding incident prediabetes or T2D (*n* = 626), we excluded 237 participants with OGTT-defined prediabetes at baseline (S4). Second, for analyses on glycemic and insulin-related traits (*n* = 792–804), we excluded 59 participants taking glucose-lowering medication and 0–12 participants with missing data on continuous traits at F4 and/or FF4 examinations, respectively.

### Bioelectrical impedance analysis

The PhA was assessed at baseline using a BIA 2000-S (DATA-INPUT GmbH, Frankfurt, Germany) and a Body Composition Analyzer TVI-10 (Danninger Medical Technology, Heidelberg, Germany) in the S3 study [21], and a BIA 2000-S (DATA-INPUT GmbH, Frankfurt, Germany) in the S4 study [4]. Both were single-frequency devices with a 50 kHz frequency and 800 μA alternating current. The BIA measurements were performed under highly standardized conditions (supplementary methods) [21]. Participants were required to abstain from meals, fluid intake, and physical activity for at least 2 h prior to the measurement. Eligible participants were instructed to empty their bladders, remove all metal objects (e.g., keys, wristwatches, jewelry), take off their stockings, and lie down in a relaxed, motionless supine position on a nonconductive surface before the measurement. The BIA measurements were performed using a tetrapolar gel-based adhesive electrode configuration, with two electrodes attached to their dominant hand and two attached to their ipsilateral foot. Analyses of intra- and inter-observer variability showed high measurement reliability, with coefficients of variation consistently below 1% [21]. BIA devices were calibrated daily, with measurements within the target values (R 500 ± 4 Ω; Xc 144 ± 4 Ω) [4]. Two repeated measurements were performed for each participant to assess consistency, requiring measurement error ≤ 1% (R ± 5 Ω; Xc ± 2 Ω). If the criteria were unmet, two additional measurements were taken. The PhA was calculated from R and Xc [5]: PhA [^o^] = $({\rm{arctangent}}\left(\frac{{\rm{Xc}}}{{\rm{R}}}\right)\times \frac{{180}^{0}}{\pi })$. Height-standardized R and Xc (R/H; Xc/H) were calculated for comparisons.

### Outcomes

In S3/S4 studies, prevalent T2D was defined as known T2D at baseline, while incident T2D was defined as known T2D occurring throughout the follow-up period until 2016. Participants with known T2D were identified by self-report and were subsequently confirmed by the responsible physicians or medical chart review.

In S4/F4/FF4 studies, participants without known diabetes received a standard 75 g OGTT after fasting for ≥ 8 h at each visit to ascertain prediabetes and previously undiagnosed T2D, based on the 1999/2006 World Health Organization criteria [22, 23]. Specifically, (1) normoglycemia was defined as having fasting glucose < 6.1 mmol/l and 2-h glucose < 7.8 mmol/l; (2) OGTT-defined prediabetes was identified as having fasting glucose ≥ 6.1 mmol/l but < 7.0 mmol/l and 2-h glucose < 7.8 mmol/l (isolated impaired fasting glucose [i-IFG]) or fasting glucose < 6.1 mmol/l and 2-h glucose ≥ 7.8 mmol/l but < 11.1 mmol/l (isolated impaired glucose tolerance [i-IGT]) or both i-IFG and i-IGT; (3) newly OGTT-defined T2D was ascertained as having fasting glucose ≥ 7.0 mmol/l or 2-h glucose ≥ 11.1 mmol/l; and (4) incident prediabetes/T2D was defined among those having normoglycemia at baseline (S4) as newly OGTT-defined prediabetes or newly OGTT-defined T2D ascertained at F4 and/or FF4 visits or known T2D ascertained during follow-up until the end of the FF4 study (2013–2014). The combined outcome of prediabetes or T2D (prediabetes/T2D) was analyzed due to the limited sample size within this subsample. Continuous traits, including fasting glucose, 2-h glucose, updated homeostatic model assessment of insulin resistance (HOMA2-IR), updated homeostatic model assessment of beta cell function (HOMA2-B), and glycated hemoglobin A1c (HbA1c) were measured at all three visits (Supplementary Methods).

### Covariates

Data on age (years), sex (men; women), smoking status (never; former; current), alcohol consumption (no; moderate; heavy), physical activity (> 2 h/week; 1–2 h/week; < 1 h/week; none), healthy eating score (score, ranging from 3 to 27), use of lipid-lowering medication (no; yes), use of diuretics (no; yes), and parental history of diabetes (no; unknown; yes) were collected using standardized questionnaires [24–26]. Height (cm), weight (kg), waist circumference (WC, cm), waist-hip ratio (WHR), BMI (kg/m^2^), hypertension (no; yes), high-density lipoprotein cholesterol (HDL-C, mmol/l), triglycerides (mmol/l), estimated glomerular filtration rate (e-GFR, ml/min/1.73 m^2^), uric acid (μmol/l), albumin (g/l), high-sensitivity C-reactive protein (hs-CRP, mg/l), and N-terminal pro-B-type natriuretic peptide (NT-proBNP, pg/ml) were measured using standard methods. Body fat percentage (BFP, %), fat-free mass (FFM, kg) [27], FFM index (FFMI, by height squared, kg/m^2^), SMM (kg) [28], and SMM index (SMMI, by height squared, kg/m^2^) were derived from BIA (Supplementary Methods).

### Statistical methods

Data analyses were performed using R (v4.4.3) [29]. Continuous variables were summarized as mean ± standard deviation for normally distributed data or median (interquartile range) for skewed data, and categorical variables were presented as frequencies (percentage). Pearson correlation analyses were performed to examine correlations of the PhA with anthropometric measures, body composition, and muscle-related parameters.

#### Cross-sectional association with prevalent T2D (S3/S4)

In S3/S4 studies, multivariable binary logistic regression models were applied to investigate the cross-sectional association of the PhA with prevalent T2D at baseline to facilitate comparisons with prior studies. Odds ratios (ORs) and 95% confidence intervals (CIs) per 1-degree increase in the PhA were calculated. Sex-specific analyses were performed due to a significant interaction between sex and the PhA. Moreover, restricted cubic splines (RCS) were applied to explore potential nonlinear relationships.

#### Longitudinal association with incident T2D (S3/S4) and incident prediabetes/T2D (S4/F4/FF4)

In S3/S4 studies, multivariable Cox proportional hazard models were performed to investigate the longitudinal association of the baseline PhA with incident T2D. Hazard ratios (HRs) and 95% CIs per 1-degree increase in the PhA were calculated. Sex-specific regression and RCS were performed for comparison to the cross-sectional results, despite no significant sex interaction. Participants were also stratified by age (< 55 and ≥ 55 years) and BMI (< 35 and ≥ 35 kg/m^2^). In sensitivity analyses, we excluded 103 participants with follow-up less than 2 years to examine potential bias from undiagnosed T2D at baseline. Additionally, the Fine–Gray sub-distribution hazard model was performed to account for the competing risk of death.

In S4/F4/FF4 studies, semi-parametric interval-censored Cox regression models [30] were performed to examine the association of the baseline PhA with incident prediabetes/T2D since the exact date of outcome occurrence was unknown. HRs with 500x bootstrapping-constructed 95% CIs were reported. Sex-specific regressions were also performed for comparison.

#### Cross-sectional and longitudinal associations with continuous traits (S4/F4/FF4)

In S4/F4/FF4 studies, two-level growth models [31] (Supplementary Methods) were applied to assess the cross-sectional and longitudinal associations of the baseline PhA with glycemic and insulin-related traits using data from three time points (S4, F4, and FF4) among participants without known or newly OGTT-defined diabetes at S4.

#### Model adjustment

Potential covariates were determined based on prior literature and data availability. All models were adjusted for age and sex. In the S3/S4 studies, study and fasting status were additionally included regardless of statistical significance to account for potential inter-study differences and PhA variations related to fasting status. Model 1 was adjusted for age, sex, fasting status (for S3/S4 only), and study (for S3/S4 only). Model 2 was further adjusted for WC, smoking status, alcohol consumption, physical activity, and healthy eating score. Model 3 was additionally adjusted for hypertension, triglycerides (for S4/F4/FF4 only), HDL-C, e-GFR, uric acid, use of lipid-lowering medication, and parental history of diabetes.

In sensitivity analyses, using available data, we (1) adjusted for WHR, BMI, or BFP to substitute WC to assess the impact of overall adiposity; (2) adjusted for FFM, FFMI, SMM, and SMMI to assess the impact of body composition and muscle mass; (3) adjusted for triglycerides (S3/S4), albumin, hs-CRP, and NT-proBNP to control for metabolic, nutritional, inflammatory, and cardiac influences on the PhA; and (4) adjusted for intake of diuretics due to their influence on fluid balance.

## Results

### Baseline characteristics and correlation analyses

In cross-sectional analyses (S3/S4; Table S1), 324 (men: *n* = 189; women: *n* = 135) out of 7728 (men: *n* = 3862; women: *n* = 3866) participants had known T2D at baseline. Men had higher PhA values compared to women (*p* < 0.001). Participants with prevalent T2D had lower PhA (both: *p* < 0.001; men: *p* < 0.001; women: *p* < 0.001), R/H (both: *p* < 0.001; men: *p* = 0.231; women: *p* < 0.001), and Xc/H (both: *p* < 0.001; men: *p* < 0.001; women: *p* < 0.001) values compared to those without in both sexes, although the differences for R/H were not significant among men.

In longitudinal analyses (S3/S4; Table 1), during a median follow-up period of 15.7 years (total person-years: 104,876), 707 (men: *n* = 407; women: *n* = 300) out of 7006 (men: *n* = 3487; women: *n* = 3519) participants without known diabetes at baseline developed T2D. Participants who developed T2D were more likely to be men, older, and have higher WC, BMI, FFM, SMM, triglycerides, uric acid, and hypertension prevalence, whereas their physical activity levels, HDL-C concentrations, and e-GFR levels were lower (all *p* < 0.001). Participants who developed T2D also showed lower R/H (*p* < 0.001) and Xc/H (*p* < 0.001) values compared to those who did not, while no significant difference in PhA values (*p* = 0.271) was observed at baseline. In correlation analyses (S3/S4; Fig. S4) in the total group, the PhA was negatively correlated with R/H (*r* = −0.42), and BFP (*r* = −0.43), but was positively correlated with Xc/H (*r* = 0.31), FFM (*r* = 0.47), FFMI (*r* = 0.45), SMM (*r* = 0.49), and SMMI (*r* = 0.51). The PhA was consistently negatively correlated with age (< 55 years: *r* = −0.17; ≥ 55 years: *r* = −0.36) and showed BMI-dependent correlations, with positive correlations at a BMI < 35 kg/m² (*r* = 0.11) and inverse correlations at a BMI ≥ 35 kg/m^2^ (*r* = −0.11) (Table S2).

**Table 1:** Baseline characteristics of the study population for longitudinal analyses in the KORA S3/S4 and S4/F4/FF4 studies.

Characteristics	S3/S4 studies		S4/F4/FF4 studies	
	Incident T2D^a^		Incident prediabetes/T2D^b^	
	Non-cases	Cases	Non-cases	Cases
*N*	6299	707	375	251
S4 study, *n* (%)	3323 (52.8)	333 (47.1)^**^	375 (100.0)	251 (100.0)
Person-year, years	98,862	6014		
Age (years)	47.8 (13.7)	56.0 (11.0)^***^	62.6 (5.4)	63.0 (5.3)
Men, *n* (%)	3080 (48.9)	407 (57.6)^***^	166 (44.3)	130 (51.8)
Fasting status, yes, *n* (%)	1604 (25.5)	255 (36.1)^***^	375 (100.0)	251 (100.0)
Waist circumference (cm)	103.4 (8.3)	109.2 (9.2)^***^	91.5 (11.0)	94.6 (11.0)^**^
WHR	0.9 (0.09)	0.9 (0.08)^***^	0.9 (0.1)	0.9 (0.1)^**^
BMI (kg/m^2^) ^c^	26.5 (4.2)	30.7 (4.6)^***^	27.1 (3.8)	28.3 (4.0)^***^
BFP (%) ^c^	31.5 (7.1)	35.1 (6.7)^***^	33.6 (6.7)	33.8 (7.1)
FFM (kg)^c^	51.5 (10.2)	55.4 (10.1)^***^	49.5 (9.4)	51.1 (9.3)^**^
FFMI (kg/m^2^)^c^	18.0 (2.3)	19.8 (2.3)^***^	17.8 (2.1)	18.5 (2.2)^***^
SMM (kg)^c^	29.7 (6.6)	30.7 (6.5)^***^	23.7 (6.2)	24.4 (6.1)
SMMI (kg/m^2^)^c^	10.3 (1.5)	10.9 (1.6)^***^	8.5 (1.5)	8.8 (1.6)^**^
Smoking status, *n* (%)		^**^		
never	2692 (42.7)	285 (40.3)	181 (48.3)	127 (50.6)
former	1945 (30.9)	252 (35.6)	141 (37.6)	93 (37.1)
current	1662 (26.4)	170 (24.0)	53 (14.1)	31 (12.4)
Alcohol consumption, *n* (%)				
no	1811 (28.8)	206 (29.1)	90 (24.0)	61 (24.3)
moderate	3154 (50.1)	371 (52.5)	211 (56.3)	140 (55.8)
heavy	1334 (21.2)	130 (18.4)	74 (19.7)	50 (19.9)
Physical activity, *n* (%)		^***^		
>2 h/week	1324 (21.0)	101 (14.3)	78 (20.8)	49 (19.5)
1–2 h/week	1746 (27.7)	146 (20.7)	126 (33.6)	63 (25.1)
<1 h/week	1015 (16.1)	132 (18.7)	54 (14.4)	46 (18.3)
none	2214 (35.1)	328 (46.4)	117 (31.2)	93 (37.1)
Healthy eating score	15.2 (3.6)	15.3 (3.5)	16.2 (3.5)	16.3 (3.6)
Hypertension, yes, *n* (%)	2149 (34.1)	455 (64.4)^***^	141 (37.6)	132 (52.6)^***^
HDL-C (mmol/l)	1.5 (0.4)	1.3 (0.4)^***^	1.6 (0.4)	1.5 (0.4)^**^
Triglycerides (mmol/l) ^c^	1.4 [1.0, 2.1]	1.9 [1.3, 2.9]^***^	1.2 [0.9, 1.6]	1.3 [1.0, 1.8]^**^
eGFR (ml/min/1.73 m^2^)	98.2 (16.7)	92.5 (16.3)^***^	83.7 (11.2)	83.5 (12.1)
Uric acid (μmol/l)	302.9 (83.0)	350.4 (85.4)^***^	310.7 (69.4)	339.3 (88.8)^***^
Albumin (g/l)^c^	41.5 (4.9)	41.1 (4.7)^**^	38.3 (4.2)	38.1 (3.6)
hs-CRP (mg/l)^c^	1.1 [0.5–2.5]	2.5 [1.2–4.7]^***^	1.2 [0.6, 2.7]	1.7 [0.8, 3.2]^**^
NT-proBNP (pg/ml)^c^	46.9 [25.2–85.3]	52.4 [26.4–113.6]^**^	71.4 [43.9, 124.6]	85.9 [51.7, 143.9] ^**^
Intake of lipid-lowering medication, yes, *n* (%)	234 (3.7)	46 (6.5)^***^	33 (8.8)	29 (11.6)
Intake of diuretics, yes, *n* (%)	354 (5.6)	111 (15.7)^***^	33 (8.8)	31 (12.4)
Parental history of diabetes, *n* (%)		^***^		^**^
no	4134 (65.6)	342 (48.4)	238 (63.5)	137 (54.6)
unknown	932 (14.8)	157 (22.2)	68 (18.1)	45 (17.9)
yes	1233 (19.6)	208 (29.4)	69 (18.4)	69 (27.5)
PhA (°)	6.2 (0.8)	6.2 (0.8)	5.7 (0.7)	5.9 (0.8)^**^
R/H (Ω/m)	315.1 (56.3)	293.8 (50.5)^***^	323.0 (55.3)	314.7 (53.6)
Xc/H (Ω/m)	33.9 (5.8)	31.8 (5.5)^***^	32.2 (5.7)	32.4 (6.1)

Continuous variables with normal distribution are presented as mean (standard deviation) and with skewed distribution as median [Q1, Q3]. Categorical variables are shown as *n* (%). *T*-test or Kruskal Wallis test and Chi-square test were applied for comparison of continuous and categorical variables among non-cases and cases, respectively.

*T2D* type 2 diabetes, *WHR* waist-hip ratio, *BMI* body mass index, *BFP* body fat percentage, *FFM* fat-free mass, *FFMI* FFM index (by height squared), *SMM* skeletal muscle mass, *SMMI* SMM index (by height squared), *HDL-C* high-density lipoprotein cholesterol, *eGFR* estimated glomerular filtration rate, *hs-CRP* high-sensitivity C-reactive protein, *NT-proBNP* N-terminal pro-B-type natriuretic peptide, *PhA* phase angle, *R/H* resistance by height, *Xc/H* reactance by height.

^***^indicates *p* < 0.001, ^**^ indicates *p* < 0.05.

^a^Incident T2D (S3/S4): known T2D ascertained during follow-up until 2016.

^b^Incident prediabetes/T2D (S4/F4/FF4): oral glucose tolerance test [OGTT]-defined prediabetes or OGTT-defined T2D identified at follow-up visits (F4 or FF4) or known T2D ascertained during follow-up until the end of FF4.

^c^Participants number in the S3/S4 studies: WHR (*n* = 7005); BMI, BFP, FFM, FFMI (*n* = 6999); SMM and SMMI (*n* = 7004); triglycerides (*n* = 4795); albumin (*n* = 6920); hs-CRP (*n* = 6909); and NT-proBNP (*n* = 4718).

In the S4/F4/FF4 studies (Table 1), similar differences in baseline characteristics were observed as in the S3/S4 studies among participants who developed prediabetes/T2D compared to those who remained normoglycemic; however, incident cases showed significantly higher PhA values compared to non-cases at baseline (*p* = 0.001). Participant characteristics for continuous traits are provided in Table S3.

### Cross-sectional associations with prevalent T2D (S3/S4)

In cross-sectional analyses (Table 2), no significant associations of the PhA (per 1-degree increase) with prevalent T2D were observed in the total group (Model 3, OR [95% CI]: 0.84 [0.69–1.03]) at baseline, while interaction with sex was present (Model 3, *p*_sex-interaction_ = 0.004). The PhA (per 1-degree increase) showed an inverse association with prevalent T2D in men (Model 3, OR [95% CI]: 0.72 [0.56–0.93]) with a linear pattern (Fig. S5A), while in women, no significant associations were found in the fully adjusted model (Model 3, OR [95% CI]: 1.12 [0.81–1.55]) (Table 2).

**Table 2:** Cross-sectional association of the PhA with prevalent T2D at baseline in the KORA S3/S4 studies.

	Both	Men	Women	*p*_sex-interaction_
*N*	7728	3862	3866	
Cases	324	189	135	
OR [95% CI]				
Model 1	0.96 [0.80–1.16]	0.76 [0.60–0.97]^**^	1.38 [1.03–1.84]^**^	0.002
Model 2	0.89 [0.73–1.08]	0.77 [0.61–0.99]^**^	1.14 [0.83–1.55]	0.012
Model 3	0.84 [0.69–1.03]	0.72 [0.56–0.93]^**^	1.12 [0.81–1.55]	0.004

Cases in the cross-sectional analyses refer to participants with known T2D ascertained at baseline. The OR and 95% CI are per 1-degree increase of the PhA at baseline (S3/S4).

Model 1: adjusted for age, sex (only for both), study, and fasting status.

Model 2: adjusted for variables in model 1 plus waist circumference, smoking status, alcohol consumption, physical activity, and healthy eating score.

Model 3: adjusted for variables in model 2 plus hypertension, high-density lipoprotein cholesterol, estimated glomerular filtration rate, uric acid, intake of lipid-lowering medication, and parental history of diabetes.

*PhA* phase angle, *T2D* type 2 diabetes, *OR* odds ratio, *CI* confidence interval.

^**^ indicates *p* < 0.05.

### Longitudinal associations with prediabetes and/or T2D (S3/S4 and S4/F4/FF4)

Our longitudinal analyses (S3/S4; Table 3) revealed that the baseline PhA (per 1-degree increase) was positively associated with incident T2D in the total group (Model 3, HR [95% CI]: 1.37 [1.21–1.54]). Likewise, in both sexes, a higher PhA was consistently associated with an increased risk of incident T2D (Model 3, HR [95% CI] per 1-degree increase: 1.42 [1.21–1.68] for men and 1.32 [1.10–1.58] for women), with linear trends (Fig. S5B). No significant differences were observed for the positive association of the PhA with incident T2D across age subgroups; however, the association became non-significant among participants with a BMI ≥ 35 kg/m^2^ (Table S4). The positive association remained significant after excluding participants with a follow-up time < 2 years, after accounting for the competing risk of death, and with alternative model adjustments. However, after adjusting for FFMI, the association was not significant anymore (Table S5).

**Table 3:** Longitudinal association of the baseline PhA with incident T2D or incident prediabetes/T2D.

	Both	Men	Women	*p*_sex-interaction_
S3/S4 studies: Incident T2D^a^				
*N*	7006	3487	3519	
Cases	707	407	300	
Person-years	104,876	50,827	54,049	
HR [95% CI]				
Model 1	1.52 [1.35–1.70]^***^	1.47 [1.25–1.73]^***^	1.58 [1.34–1.87]^***^	0.419
Model 2	1.45 [1.29–1.63]^***^	1.52 [1.29–1.78]^***^	1.40 [1.17–1.67]^***^	0.800
Model 3	1.37 [1.21–1.54]^***^	1.42 [1.21–1.68]^***^	1.32 [1.10–1.58]^**^	0.372
S4/F4/FF4 studies: Incident prediabetes/T2D ^b^				
*N*	626	296	330	
Cases	251	130	121	
HR [95% CI]				
Model 1	1.40 [1.13–1.74]^**^	1.60 [1.09–2.35]^**^	1.29 [0.98–1.71]	0.320
Model 2	1.38 [1.11–1.72]^**^	1.55 [1.03–2.31]^**^	1.24 [0.91–1.69]	0.409
Model 3	1.33 [1.07–1.67]^**^	1.62 [1.07–2.47]^**^	1.22 [0.86–1.72]	0.441

The HR and 95% CI are per 1-degree increase of the baseline PhA.

Model 1: adjusted for age, sex (only for both), study (for S3/S4), and fasting status (for S3/S4).

Model 2: adjusted for variables in model 1 plus waist circumference, smoking status, alcohol consumption, physical activity, and healthy eating score.

Model 3: adjusted for variables in model 2 plus hypertension, high-density lipoprotein cholesterol, log_e_-transformed triglycerides (for S4/F4/FF4), estimated glomerular filtration rate, uric acid, intake of lipid-lowering medication, and parental history of diabetes.

*PhA* phase angle, *T2D* type 2 diabetes, *HR* hazard ratio, *CI* confidence interval.

^***^ indicates *p* < 0.001, ^**^ indicates *p* < 0.05.

^a^Incident T2D (S3/S4): participants with known T2D ascertained during follow-up until 2016.

^b^Incident prediabetes/T2D (S4/F4/FF4): participants with OGTT-defined prediabetes or OGTT-defined T2D identified at follow-up visits (F4 or FF4) during follow-up until the end of FF4 or known T2D ascertained during follow-up until the end of FF4.

In the S4/F4/FF4 subsample (Table 3), the baseline PhA (per 1-degree increase) was also positively associated with the combined outcomes of incident prediabetes/T2D in the total group (Model 3, HR [95% CI]: 1.33 [1.07–1.67]), but we did not observe a significant sex-interaction (Model 3, *p*_sex-interaction_ = 0.441). In sex-specific analyses, the positive association was significant in men (Model 3, HR [95% CI]: 1.62 [1.07–2.47]), but non-significant in women (Model 3, HR [95% CI]: 1.22 [0.86–1.72]), possibly due to power limitations (Table 3). The positive association in the total group remained robust after alternative adjustments but was again attenuated upon adjustment for FFMI (Table S5).

### Cross-sectional and longitudinal associations with continuous traits (S4/F4/FF4)

**Fig. 1:**
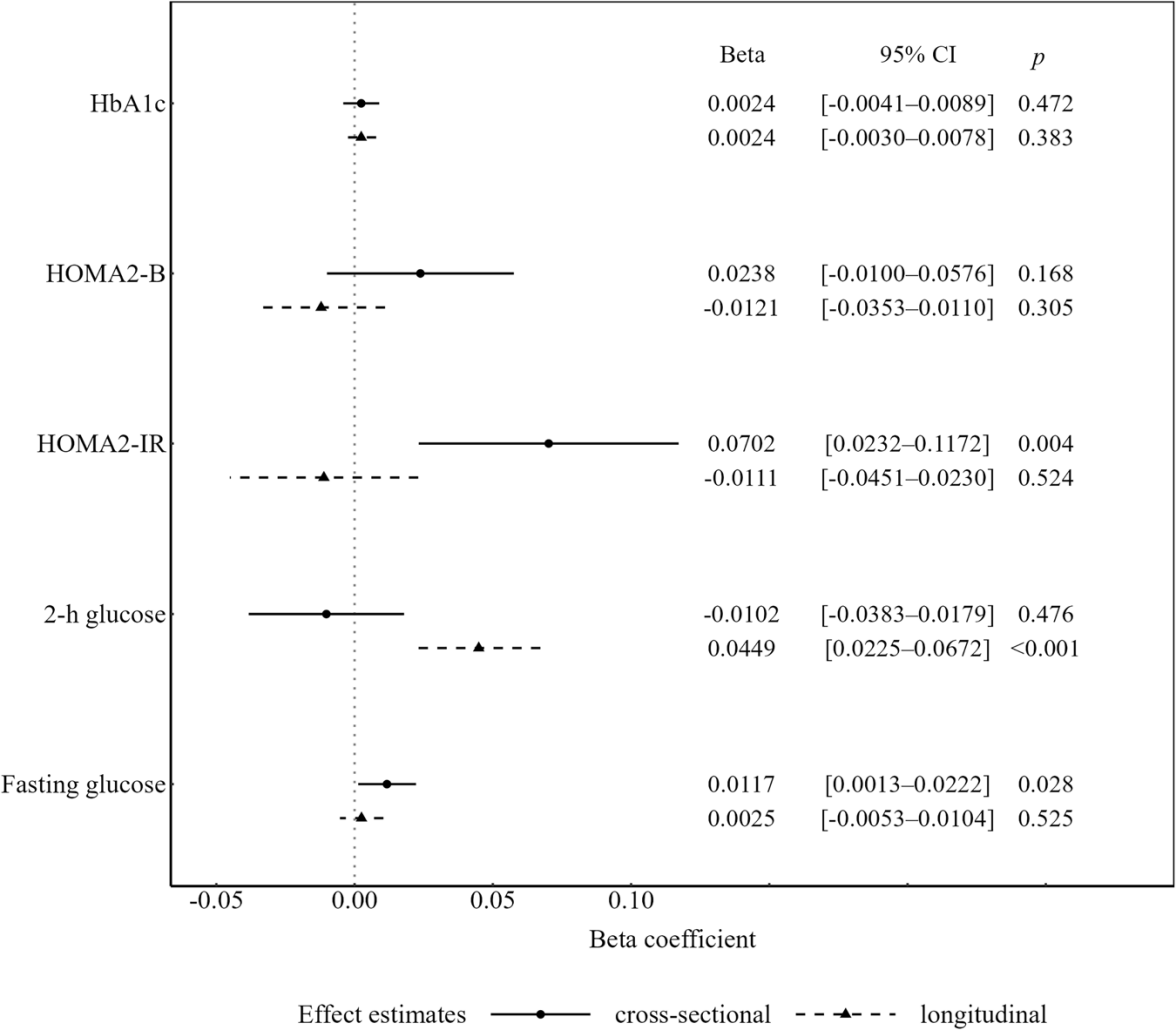
Associations of the PhA with glycemic and insulin-related traits in participants without diabetes at baseline in the KORA S4/F4/FF4 studies. PhA phase angle, 2-h glucose 2-h glucose, HOMA2-IR updated homeostatic model assessment of insulin resistance, HOMA2-B updated homeostatic model assessment of beta-cell function, HbA1c glycated hemoglobin A1c. Analyses were conducted in the KORA S4/F4/FF4 studies among participants without known or newly OGTT-defined diabetes at S4 (*n* = 792–804). Cross-sectional (between-participant) effects refer to the association between the baseline PhA and variations of the five continuous traits at baseline. Longitudinal (within-participant) effects refer to the association between the baseline phase angle and changes in the five continuous traits over a 10-year period. Effects are shown as beta coefficients with 95% confidence intervals for cross-sectional (circle and solid line) and longitudinal (triangle and dashed line) associations per 1-degree increase of the baseline PhA. Models (Model 3) were adjusted for age, sex, waist circumference, smoking status, alcohol consumption, physical activity, healthy eating score, hypertension, high-density lipoprotein cholesterol, triglycerides, estimated glomerular filtration rate, uric acid, intake of lipid-lowering medication, and parental history of diabetes.

In the S4/F4/FF4 studies, among participants without known or newly OGTT-defined T2D at baseline (Fig. 1), the baseline PhA (per 1-degree increase) was positively associated with fasting glucose (cross-sectional effect; beta [95% CI]: 1.2% [0.1–2.2%]) and HOMA2-IR (beta [95% CI]: 7.0% [2.3–11.7%]) and with the rate of change in 2-h glucose (longitudinal effect; beta [95% CI]: 4.5% [2.3–6.7%] over 10 years). Full model results are provided in Table S6.

## Discussion

### Main findings

Among men and women without diabetes at baseline, those with higher baseline PhA values at 50 kHz had a higher risk of developing T2D. Higher PhA values were further associated with an increased risk of developing prediabetes/T2D in a subgroup of normoglycemic participants at baseline. Supporting this, higher PhA values were also associated with elevated fasting glucose and HOMA2-IR cross-sectionally and with increased 2-h glucose longitudinally among participants without diabetes at baseline. In contrast, higher PhA values were linked to a lower risk of prevalent T2D in men but not in women at baseline.

### Overview of prior studies

Longitudinal studies on the association of the PhA with incident T2D have not been conducted before. However, our cross-sectional findings align with the previously largest study with data from 1085 Malaysian adults aged ≥ 55 years, as Mat et al. [13] reported that the PhA was inversely associated with prevalent T2D in men, whereas no significant association was found in women. Most prior studies reported lower PhA values at 50 kHz in individuals with diabetes compared to controls [14, 15, 17, 32–34]. Notably, several of these studies did not investigate sex differences, partly due to limited sample sizes [32–34]. For instance, Buscemi et al. [14] reported significantly lower PhA values among 499 outpatients aged 18–65 years with type 1 diabetes (T1D) or T2D compared to 113 healthy volunteers with normal glucose tolerance in Italy. In contrast, two other studies reported higher PhA values in men and women with T2D compared to controls [16, 35]. In the study of Buffa et al. [16], older adults aged 60–84 years with T2D who were not treated with insulin showed higher PhA values compared to healthy BMI-matched groups. Likely, most participants in their study were in an early rather than severe stage, since they did not receive insulin. Salis et al. [35] also observed higher PhA values in both the controlled and uncontrolled diabetes groups compared to the non-diabetes group. Yet, the differences between groups were not significant, possibly due to limited sample sizes. Persons in the diabetes group were younger, possibly explaining the higher PhA values since results were not adjusted for age [35]. Higher PhA values were also previously observed among 1399 adults with obesity (BMI ≥ 28 kg/m^2^) compared with 330 overweight adults (BMI 24–27.9 kg/m^2^) [36], and among 682 adults with overweight and obesity (BMI ≥ 24 kg/m^2^) with nonalcoholic fatty liver disease compared to 271 without [37]. Both obesity and non-alcoholic fatty liver disease are strongly linked to insulin resistance (IR), which could fit the observed positive association between the PhA and HOMA2-IR among persons without diabetes in the present study. Notably, several previous studies relied on unadjusted comparisons without consideration of potential confounders [14, 15, 17], and some did not report the BIA measurement devices or frequency [13, 16, 33, 35], limiting comparability across studies.

### Potential mechanisms

The underlying cellular mechanisms linking the PhA to diabetes are not yet fully understood. The seemingly contradictory cross-sectional and longitudinal findings could suggest a stage-dependent association throughout the course of T2D progression. The association of higher baseline PhA with incident T2D might indicate that higher PhA reflects early metabolic dysfunction preceding overt glycemic deterioration that is associated with subsequent risk of diabetes development, whereas the association of lower PhA with prevalent T2D could indicate long-term glycemic deterioration. The cross-sectional findings further suggest a potential sex-specific association after diabetes onset.

Early metabolic changes preceding the clinical onset of type 2 diabetes, such as IR and low-grade inflammation, may influence the PhA by altering cellular membrane properties (e.g., permittivity), fluid distribution, and tissue conductivity. IR is a central pathophysiological feature of T2D, which precedes its clinical manifestation, initially triggers compensatory hyperinsulinemia with anabolic effects, whereas levels of insulin decrease during the progression of diabetes over time[38]. We previously observed that the PhA was inversely associated with insulin-like growth factor binding protein 2 (IGFBP2) [4], known to be inversely related to IR and T2D risk [39], which may support the positive association of the PhA with IR and incident T2D. Lower IGFBP2 could reduce suppression of free IGF-1, potentially promoting cell proliferation (reflected by higher BCM and PhA) [4] and adipogenesis, especially in visceral adipocytes, and modulation of insulin sensitivity [40, 41]. Supporting this, a positive cross-sectional association of the PhA with HOMA2-IR was observed in the present study. At cellular levels, higher PhA values may reflect more muscle cells or adipose cells^42^. Specifically, higher proportions of type I muscle fibers are predominantly oxidative with quantities of large mitochondria and water [42], likely reflecting a metabolically active state during the early compensatory stage of glucose dysregulation.

Higher PhA values may also reflect excess body fat, especially inter- and intramuscular fat infiltration, which has also been associated with IR and increased risk of T2D [7]. We observed attenuated effect estimates regarding associations of the PhA with risks of prediabetes and/or T2D after adjusting for FFMI, suggesting that the positive effect of the PhA on the risk of prediabetes and/or T2D may be partially attributable to a high FFMI among persons at high risk of prediabetes and/or T2D. Albeit high SMM or high FFM is often considered to be beneficial rather than detrimental in terms of glucose homeostasis, a review by Perreault et al. [43] has challenged this general assumption and summarized several studies demonstrating that high FFM is associated with altered glucose homeostasis. Specifically, intramuscular fat accumulation may determine impaired insulin sensitivity, and a higher FFMI might contribute to IR, as reported in different populations [44, 45]. This is in line with the present study, where a high FFMI was associated with a greater risk of developing prediabetes and/or T2D. Future research should further explore the role of FFMI in this relationship and other possible pathways. Additionally, the positive association of the PhA with incident T2D was attenuated among individuals with a BMI ≥ 35 kg/m². This is likely due to excessive fat accumulation and expansion of ECW in individuals with higher BMI values [46], which may affect PhA measurements. Thus, caution is needed when interpreting the association of the PhA with diabetes in individuals with severe obesity.

In contrast, cross-sectional results observing lower PhA values among individuals with prevalent T2D might reflect metabolic impairments or adverse cellular changes, such as inflammation and oxidative stress, impaired cellular integrity, and hyperglycemia-induced osmotic pressure changes followed by cellular water shifts (elevated ECW/ICW) [14, 17, 18] that progress following the onset of diabetes. Especially individuals with longstanding and poorly controlled diabetes are more likely to experience these severe adverse changes. These changes, along with increased adiposity and accelerated loss of SMM or quality decline, may manifest as lower PhA values [9, 17, 47]. Supporting this, using cross-sectional data from three Korean clinics (*n* = 217), Jun et al. [18] reported a steeper decline in the PhA with age among individuals with T1D/T2D than controls, with the lowest PhA values observed in those with longer disease duration. Lower PhA values were also found in individuals with diabetes-related complications, who typically have long-term diabetes [48, 49].

The physiological basis of the observed sex differences in the cross-sectional association of the PhA with prevalent diabetes remains unclear, as available research is limited. Mat et al. [13] suggested that the lack of association among women might be explained by participant heterogeneity. Our cross-sectional analyses also confirmed that women had higher body fat but lower SMM than men, suggesting that differences in body composition may contribute to the observed sex-specific patterns. Additionally, sex differences exist in metabolic dysfunction, including glucose and lipid metabolism, effects of sex hormones, genetic factors, inflammation, and diabetes treatment and adherence [50–52], which may also explain the observed sex differences. However, we could not further explore this due to the limited number of prevalent cases in our study population.

### Limitations

Despite the prospective population-based design and the large sample size, which enabled longitudinal analyses, the present study was limited by investigating only PhA values at baseline. Second, single-frequency BIA devices were used, which might be impacted by hydration status. Although several factors were considered, a lack of direct assessment of hydration status limits the accuracy of raw bioimpedance parameters. Third, a relatively short pre-measurement fasting period (≥ 2 h) may not have fully accounted for acute postprandial changes in fluid distribution, potentially influencing BIA-derived parameters. Fourth, while the biological meaning of the PhA is not fully clarified, it reflects a composite of BCM, cellular integrity, fluid distribution, and biophysical properties such as tissue resistivity, membrane capacitance, and geometric factors (e.g., membrane thickness and size, conductive path length, and cross-sectional area). Thus, the PhA represents an indirect and integrated marker of physiological status rather than a measure of a single biological process. Fifth, despite adjusting for multiple factors, potential confounders may have been missed. Sixth, there is a risk of FFM overestimation and BFP underestimation in individuals with excess adipose tissue due to altered hydration and body water distribution [53]. Thus, the PhA should be interpreted with caution in participants with severe obesity, as it is influenced by fluid distribution. Seventh, although under similar conditions, predictive equations of FFM and SMM were developed based on different BIA devices; therefore, absolute values of these indices may not be directly interchangeable. Future studies are warranted to validate and systematically evaluate these device-specific differences against reference (gold-standard) methods, including dual-energy X-ray absorptiometry or preferably four-compartment models for fat mass and FFM, and tissue-level imaging techniques such as magnetic resonance imaging or computed tomography for SMM. Eighth, we included individuals living in Germany with predominantly Caucasian ancestry, restricting its generalizability.

The PhA has the advantages of simplicity, non-invasiveness, and being free from equation-inherent errors and necessary assumptions. It also reflects cellular-level alterations that are not fully captured by conventional anthropometric measures. Thus, the PhA may serve as a complementary marker of early metabolic alterations associated with later T2D development in both clinical and population-based settings, particularly in resource-limited settings. However, current evidence remains limited. Future longitudinal studies with repeated BIA assessments are necessary to examine whether temporal changes in the PhA and other bioimpedance-derived parameters are associated with the development or progression of prediabetes and T2D. Moreover, future studies with repeated BIA measurements across diverse populations, formal prediction models, and the establishment of sex-, age-, and population-specific reference ranges are necessary to improve interpretability and assess the incremental diagnostic or prognostic value beyond established markers for prediabetes and T2D.

In conclusion, our findings suggest a stage-dependent link between PhA values and derangements in glucose metabolism. While higher PhA values could be an indicator of IR during pre-diabetic stages and therefore an increased risk of developing T2D, long-standing diabetes may lead to lower PhA values. Future longitudinal studies, examining changes in PhA values over time in persons with and without diabetes, are warranted to further clarify these first longitudinal results and the underlying mechanisms.

## Supplementary information

SUPPLEMENTAL MATERIAL

## Data Availability

The data from this study are not publicly available due to data protection regulations and restrictions imposed by the Ethics Committee of the Bavarian Chamber of Physicians to protect participant privacy. However, data can be accessed upon request through project agreements with KORA (https://helmholtz-muenchen.managed-otrs.com/external). The code generated during the current study is available from the corresponding author on reasonable request.
