# Supplementary material for: Association of the phase angle with type 2 diabetes and related traits: results from two prospective KORA studies
Source: Nutr Diabetes. 2026 May 9;16:11. doi: 10.1038/s41387-026-00425-x (PMC13157506; doi:10.1038/s41387-026-00425-x)
Supplement: Supplementary file 1 — SUPPLEMENTAL MATERIAL [file 41387_2026_425_MOESM1_ESM.docx]

**Supplementary materials**

**Association of the phase angle with type 2 diabetes and related traits: results from two prospective KORA studies**

Feiling Ai^1,2^, Marie-Theres Huemer^1^, Wolfgang Rathmann^3,4^, Michael Roden^4,5,6^, Christian Herder^4,5,6^, Tanja Zeller^7,8^, Wolfgang Koenig^9,10,11^, Jana Nano^1,12^, Michael Drey^13^, Annette Peters^1,2,10,14^, Barbara Thorand^1,2,14*^

^1^Institute of Epidemiology, Helmholtz Zentrum München, German Research Center for Environmental Health (GmbH), Neuherberg, Germany;

^2^Institute for Medical Information Processing, Biometry, and Epidemiology (IBE), Faculty of Medicine, LMU Munich, Pettenkofer School of Public Health, Munich, Germany;

^3^Institute for Biometrics and Epidemiology, German Diabetes Center, Leibniz Center for Diabetes Research at Heinrich Heine University Düsseldorf, Düsseldorf, Germany;

^4^German Center for Diabetes Research (DZD), Partner Düsseldorf, Neuherberg, Germany;

^5^Department of Endocrinology and Diabetology, Medical Faculty and University Hospital Düsseldorf, Heinrich Heine University Düsseldorf, Düsseldorf, Germany;

^6^Institute for Clinical Diabetology, German Diabetes Center, Leibniz Center for Diabetes Research at Heinrich Heine University Düsseldorf, Düsseldorf, Germany;

^7^Institute for Cardiogenetics, University Heart Center Lübeck, University Hospital Schleswig-Holstein, University of Lübeck, Lübeck, Germany

^8^German Center for Cardiovascular Research (DZHK), Partner Site Hamburg, Lübeck, Kiel, Lübeck, Germany;

^9^German Heart Center, TUM University Hospital, Munich, Germany;

^10^German Center for Cardiovascular Research (DZHK), Partner Site Munich Heart Alliance, Munich, Germany;

^11^Institute of Epidemiology and Medical Biometry, University of Ulm, Ulm, Germany;

^12^Department of Radiation Oncology, Klinikum rechts der Isar, Technical University of Munich (TUM), Munich, Germany;

^13^Department of Medicine IV, LMU University Hospital, LMU Munich, Germany;

^14^German Center for Diabetes Research (DZD), Partner München-Neuherberg, Neuherberg, Germany.

**^*^ Corresponding author:** Prof. Dr. Barbara Thorand

Email: barbara.thorand@helmholtz-munich.de

Institute of Epidemiology, Helmholtz Zentrum München, German Research Center for Environmental Health (GmbH), Ingolstädter Landstraße 1, 85764 Neuherberg, Germany

Contents

[Supplementary methods 3](#_Toc226624084)

[1. Measurement of bioelectrical impedance analysis 3](#_Toc226624085)

[2. Measurement of glycemic and insulin-related traits 5](#_Toc226624086)

[3. Measurement and definitions of baseline covariates 7](#_Toc226624087)

[4. Two-level growth models 10](#_Toc226624088)

[5. Main R packages 11](#_Toc226624089)

[References 11](#_Toc226624090)

[Supplementary figures 13](#_Toc226624091)

[Figure S1 Overview of the general KORA studies 13](#_Toc226624092)

[Figure S2 Flowchart of the study population in the KORA S3/S4 studies 14](#_Toc226624093)

[Figure S3 Flowchart of the study population in the KORA S4/F4/FF4 studies 15](#_Toc226624094)

[Figure S4 Correlation of the PhA with anthropometric and body composition parameters at baseline in the KORA S3/S4 studies 16](#_Toc226624095)

[Figure S5 Associations of the baseline PhA with prevalent T2D and incident T2D using restricted cubic splines in the KORA S3/S4 studies 19](#_Toc226624096)

[Supplementary tables 21](#_Toc226624097)

[Table S1 Characteristics of the study population for cross-sectional analyses in the KORA S3/S4 studies 21](#_Toc226624098)

[Table S2 Pearson correlations of the PhA with age and BMI within age and BMI groups in the KORA S3/S4 studies 23](#_Toc226624099)

[Table S3 Characteristics of the study participants with continuous glycemic and insulin-related traits in the KORA S4/F4/FF4 studies 24](#_Toc226624100)

[Table S4 Stratified analyses of the longitudinal association of the baseline PhA with incident T2D in the KORA S3/S4 studies 26](#_Toc226624101)

[Table S5 Sensitivity analyses of the longitudinal association of the baseline PhA with incident T2D in the KORA S3/S4 studies or incident prediabetes/T2D in the KORA S4/F4/FF4 studies 27](#_Toc226624102)

[Table S6 Associations of the baseline PhA with glycemic and insulin-related traits among participants without diabetes at baseline in the KORA S4/F4/FF4 studies 28](#_Toc226624103)

# Supplementary methods

## 1. Measurement of bioelectrical impedance analysis

Bioelectrical impedance analysis (BIA) is based on measuring the electrical resistance that a body opposes to an alternating electrical current.

(1) Devices:

The BIA was conducted using the following devices with a 50 kHz frequency and 800 μA current with gel-based adhesive electrodes among the eligible participants.

a. S3: a BIA 2000-S (DATA-INPUT GmbH, Frankfurt, Germany) and a Body Composition Analyzer TVI-10 (Danninger Medical Technology, Heidelberg, Germany) and inter-device correction was applied to ensure comparability of measurements within the S3 study(1).

b. S4: a BIA 2000-S (DATA-INPUT GmbH, Frankfurt, Germany)(2).

(2) Participant preparation before the measurement:

a. Pregnant women, individuals with electronic devices such as pacemakers, and individuals with amputations were excluded.

b. Participants had their last meals, fluid intake, or physical activity at least two hours ago.

c. Participants were instructed to use the restroom to empty their bladder.

d. Participants were initially asked whether they were left-handed or right-handed, and the measurement was then performed on the right side of the body for right-handed individuals and on the left side of the body for left-handed individuals.

e. Participants were instructed to remove all metal objects (keys, wristwatches, jewelry, and earrings if possible).

f. Participants were asked to take off their stockings and lie down in a supine position on a nonconductive surface with their back relaxed.

(3) Electrode placement:

New electrodes are used for each participant. The electrode attachment sites were cleaned with isopropanol to ensure good conductivity; two electrodes were connected to the dominant hand wrist and dorsum, and two electrodes were connected to the ipsilateral foot ankle and dorsum of the participants, as illustrated below:


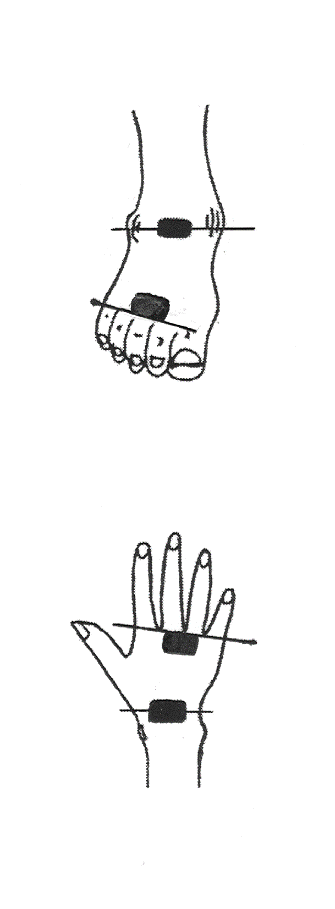


BIA measurement: electrode connection sites

(4) Measurement process:

Participants were instructed to lie down in a relaxed position, without movement, during the measurement; to hold their hands flat; and to spread their legs and arms slightly to avoid contact with other body parts. Two repeated measurements were performed, and the device compared the values. If the difference between the two measurements exceeded 5 Ω in resistance (R) or 2 Ω in reactance (Xc) (measurement error within 1% for both R and Xc), another measurement was performed.

(5) Device maintenance and calibration:

To ensure that all measuring devices provided consistent and reliable results, all the BIA devices were calibrated daily. Every day, before the first measurement and after the last use of the impedance measuring device, the battery’s charging voltage was checked, and the calibration was performed with a standard R and documented in the device folders. Target calibration values were R = 500 (±4) Ω and Xc = 144 (±4) Ω.

To improve inter-device comparability within the S3 study, the resistance measurements performed with the TVI-10 device were corrected by the addition of 0.8%. The correction factor was derived from a calibration study performed within the S3 study with triplicate measurements among 40 participants (men and women with different statures) with all three devices used in the study (two BIA-2000-S devices and one Body Composition Analyzer TVI-10 device). The results demonstrated no differences between the two BIA-2000-S devices but slightly lower resistance values for the TVI-10 device(1).

## 2. Measurement of glycemic and insulin-related traits

In the KORA S4/F4/FF4 studies, we used data measured at baseline (S4) and two follow-up examinations (F4 and FF4) for fasting glucose, 2-hour glucose (2-h glucose), updated homeostatic model assessment of insulin resistance (HOMA2-IR), updated homeostatic model assessment of beta cell function (HOMA2-B), and hemoglobin A1c (HbA1c) to evaluate glycemia, insulin resistance and secretion. The measurement details can be found in a previous publication(3).

(1) Serum glucose levels were measured using the hexokinase method in the S4 study (Gluco-quant; Roche Diagnostics, Mannheim, Germany)(4) and the F4 study (GLU Flex, Dade Behring, Deerfield, Illinois, USA)(5); while using an enzymatic, colorimetric method (GLU assay) on a Dimension Vista 1500 instrument (Siemens Healthcare Diagnostics Inc., Newark, USA) or GLUC3 assay on a Cobas c702 instrument (Roche Diagnostics GmbH, Mannheim, Germany) in the FF4 study(5, 6).

(2) Serum insulin levels were measured using a microparticle enzyme immunoassay (Abbott Laboratories, Wiesbaden, Germany) in the S4 study, using an electrochemiluminescence immunoassay on a Cobas e602 instrument (Roche Diagnostics GmbH, Mannheim, Germany) in the F4 study(5, 6), and using a solid-phase enzyme-labeled chemiluminescent immunometric assay on an Immulite 2000 systems analyzer (Siemens Healthcare Diagnostics Inc., Newark, USA) or using an electrochemiluminescence immunoassay on a Cobas e602 (Roche Diagnostics GmbH, Mannheim, Germany) in the FF4 study(5, 6).

(3) HOMA2-IR and HOMA2‐B were calculated using the HOMA2 model(7) based on the software HOMA2 Calculator V2.2.3(8):

HOMA2-IR = fasting insulin [µU/ml] × fasting glucose [mmol/l] / 22.5

HOMA2-B = 20 × fasting insulin [μU/ml] / (fasting glucose [mmol/l] $-$ 3.5)

(4) HbA1c was measured using a turbidimetric immunologic assay (Tina-quant, Roche Diagnostics GmbH, Mannheim, Germany) in the S4 study(9), using a cation-exchange high-performance liquid chromatography with the Adams HA 8160 Hemoglobin Analysis System (Arkray, distributed by A. Menarini Diagnostics, Florence, Italy) in the F4 study(5, 6), and using the Variant II Turbo HbA1c Kit-2.0 (BioRad Laboratories, Hercules, USA) in the FF4 study(5, 6).

Calibration for different methods was carried out as detailed in prior descriptions(6).

## 3. Measurement and definitions of baseline covariates

Smoking status was classified into three categories: never, former, and current. Participants who never smoked or had smoked less than 100 cigarettes in their lifetime were classified as “never smokers”, participants who smoked regularly or occasionally in the past were classified as “former smokers”, and participants who smoked at least one cigarette per day were classified as “current smokers”(10).

Alcohol consumption was classified into three categories: no, moderate, and heavy. Participants who did not consume any alcoholic beverages during the last weekend or last workday (or last Thursday if the last workday was a Friday) were assigned to the category “no alcohol consumption”, participants with an estimated alcohol consumption between > 0 – < 40 g/day for men and > 0 – < 20 g/day for women were classified as “moderate alcohol consumption” and participants with an estimated alcohol consumption ≥ 40 g/day for men and ≥ 20 g/day for women were classified as “heavy alcohol consumption”(11).

The level of physical activity was evaluated according to the duration of leisure time sport activities with four categories: > 2 h/week, 1 – 2 h/week, < 1 h/week, and (4) none.

The healthy eating score was a cumulative index reflecting adherence to the contemporary dietary recommendations of the German Nutrition Society (DGE) food pyramid at the time of assessment(12). To create the score, consumption frequency was categorized into three categories: 2 = optimal (“correct”), 1 = normal (“average”), 0 = deviating (“incorrect”), and applied to 16 selected food groups from the food frequency questionnaire. In the KORA S3/S4 studies, the healthy eating score ranged from 3 to 27 (median: 15), with higher scores indicating closer adherence to the dietary recommendations.

Hypertension was defined as blood pressure ≥ 140/90 mmHg, or the use of antihypertensive medication, given that participants were aware of being hypertensive(5). Participants were instructed to bring the original packaging of any pharmaceutical products they had taken in the last 7 days before the examination to the study center. Moreover, Anatomical Therapeutic Chemical Classification System codes (ATC) were assigned to define the medication use, such as antihypertensive, glucose-lowering, and lipid-lowering medications(13).

Body composition parameters were calculated by validated equations using the resistance and reactance measured with the BIA 2000-S (DATA-INPUT GmbH, Frankfurt, Germany) at 50 kHz and taking into consideration body height and weight. Fat-free mass was calculated from dual-energy X-ray absorptiometry (DXA)-validated 50-kHz BIA equations by Kyle et al. in 2001(14). Fat-free mass index and body fat percentage were calculated from equations created by Kyle et al. 2003(15). Skeletal muscle mass was calculated from Caucasian-specific 50-kHz BIA equations developed by Janssen et al.(16). Skeletal muscle mass index was calculated by dividing height squared.

Calculation equations:

1. Fat-free mass (kg) = −4.104 + (0.518 × height^2^/resistance) + (0.231 × weight) + (0.130 × reactance) + (4.229 × sex).
2. Fat-free mass index (kg/m²) = fat-free mass / height^2^.
3. Body fat percentage (%) = body fat / total weight × 100.
4. Skeletal muscle mass (kg) = [(height^2^/resistance × 0.401) + (sex × 3.825) + (age × -0.071)] + 5.102
5. Skeletal muscle mass index (kg/m^2^) = skeletal muscle mass / height^2^.

High-density lipoprotein cholesterol (HDL-C) was measured with enzymatic methods (CHOL Flex and AHDL Flex, Dade Behring, Marburg, Germany) and serum triglycerides with the GPO-PAP method (Dade Behring)(5).

The estimated glomerular ﬁltration rate (e-GFR, ml/min/1.73m^2^) was calculated from serum creatinine (mg/dl) using the CKD-EPI 2009 formula(17).

Serum uric acid (μmol/l) was determined with an enzymatic colorimetric reaction in the S3 study (Uric Acid PAP; Boehringer Mannheim)(18) and was assessed with the uricase method in the S4 study (Roche Diagnostics)(4).

Albumin (g/l) was measured in EDTA plasma with nephelometry using a BN II analyzer in both S3 and S4 studies (N Antiserum gegen Albumin, Fa. Siemens, Erlangen, Germany)(19, 20).

Hs-CRP (mg/l) was measured in EDTA plasma using a high-sensitivity immunoradiometric assay (IRMA) (range 0.05 – 10 mg/l) in the S3 study and using a high-sensitivity latex enhanced nephelometric assay (range > 0.16 mg/l) on a BN II analyzer in the S4 study (N Antiserum gegen CRP, Fa. Siemens, Erlangen, Germany). Both methods produced similar results when the same samples were analyzed(21).

NT-proBNP (pg/ml) was measured in EDTA plasma using an ultrasensitive single-molecule counting assay (Erenna Immunoassay System, Singulex, Alameda, CA, USA)(22).

## 4. Two-level growth models

Two-level growth models were used to assess the cross-sectional and longitudinal associations of the PhA with glycemic and insulin-related traits, including fasting glucose, 2-h glucose, HOMA2-IR, HOMA2-B, and HbA1c in the KORA S4/F4/FF4 studies.

(1) Formulas

Two-level effect estimation was performed as follows(23):

level one (within participants): $Y_{ij}=a_{i}+b_{i}\times{year}_{ij}+\epsilon_{ij}$

level two (between participants): $a_{i}=\alpha_{0}+\alpha_{1}\times{PhA}_{i}+u_{i}$

$b_{i}=\beta_{0}+\beta_{1}\times{PhA}_{i}+v_{i}$

$i$: participant, $j$: year, $\epsilon_{ij}\sim N(0, \sigma^{2})$ .

The level one effects (within-participant effects) represent the longitudinal association between the baseline PhA and changes in glycemic and insulin-related traits over time, and the level two effects (between-participant effects) represent the cross-sectional association between the PhA and individual variations of the five continuous traits at baseline.

All continuous traits underwent a natural logarithm transformation (log_e_) to ensure normal distribution, and the longitudinal effects were estimated over 10 years.

(2) Result interpretation

To display the results more comprehensively, we calculated changes in glycemic and insulin-related traits over 10 years to capture longitudinal effects (annual association $\times$ 10). Subsequently, we interpreted the effects of a one-degree change in the baseline PhA as coefficients $\times$ 100 percent change on its original scale, given that the log_e_ scale approximates a 1% change on the original values. Therefore, the longitudinal effect estimates can be understood as a one-degree difference in the PhA at baseline leading to an average β_1_ $\times$ 100 percent change in the rate of change over 10 years in the continuous traits on their original scale. Similarly, the cross-sectional effect estimates can be interpreted as a one-degree difference in individual PhA at baseline, resulting in an average $\alpha_{1}$ $\times$ 100 percent change in the continuous traits on their original scale.

## 5. Main R packages

Restricted cubic splines: package “rms” v7.0-0.

Multivariable Cox proportional hazard models: package “survival” v3.8-3.

Semi-parametric interval-censored Cox regression models: package “icenReg” v2.0.16.

Two-level growth models: package “lmerTest” v3.1-3.

## References

1. Kussmaul B, Döring A, Filipiak B. Bioelektrische Impedanzanalyse (BIA) in einer epidemiologischen Studie. Ernährungs-Umschau. 1996;43(2):46-8.

2. Huemer MT, Petrera A, Hauck SM, Drey M, Peters A, Thorand B. Proteomics of the phase angle: results from the population-based KORA S4 study. Clin Nutr. 2022;41(8):1818-26.

3. Luo H, Bauer A, Nano J, Petrera A, Rathmann W, Herder C, et al. Associations of plasma proteomics with type 2 diabetes and related traits: results from the longitudinal KORA S4/F4/FF4 Study. Diabetologia. 2023;66(9):1655-68.

4. Rathmann W, Haastert B, Icks A, Lowel H, Meisinger C, Holle R, et al. High prevalence of undiagnosed diabetes mellitus in Southern Germany: target populations for efficient screening. The KORA survey 2000. Diabetologia. 2003;46(2):182-9.

5. Sujana C, Seissler J, Jordan J, Rathmann W, Koenig W, Roden M, et al. Associations of cardiac stress biomarkers with incident type 2 diabetes and changes in glucose metabolism: KORA F4/FF4 study. Cardiovasc Diabetol. 2020;19(1):178.

6. Huth C, von Toerne C, Schederecker F, de Las Heras Gala T, Herder C, Kronenberg F, et al. Protein markers and risk of type 2 diabetes and prediabetes: a targeted proteomics approach in the KORA F4/FF4 study. Eur J Epidemiol. 2019;34(4):409-22.

7. Levy JC, Matthews DR, Hermans MP. Correct homeostasis model assessment (HOMA) evaluation uses the computer program. Diabetes Care. 1998;21(12):2191-2.

8. HOMA2 Calculator V2.2.3. Diabetes Trials Unit, University of Oxford [Available from: <https://www2.dtu.ox.ac.uk/homacalculator/>.

9. Rathmann W, Haastert B, Icks A, Löwel H, Meisinger C, Holle R, et al. High prevalence of undiagnosed diabetes mellitus in Southern Germany: target populations for efficient screening. The KORA survey 2000. Diabetologia. 2003;46(2):182-9.

10. Wacker M, Holle R, Heinrich J, Ladwig K-H, Peters A, Leidl R, et al. The association of smoking status with healthcare utilisation, productivity loss and resulting costs: results from the population-based KORA F4 study. BMC Health Services Research. 2013;13(1):278.

11. Ruf E, Baumert J, Meisinger C, Doring A, Ladwig KH, Investigators MK. Are psychosocial stressors associated with the relationship of alcohol consumption and all-cause mortality? BMC Public Health. 2014;14(1):312.

12. Winkler G, Döring A. Kurzmethoden zur Charakterisierung des Ernährungsmusters: Einsatz und Auswertung eines Food-Frequency-Fragebogens. Ernährungs-Umschau. 1995;42:289-91.

13. Laxy M, Knoll G, Schunk M, Meisinger C, Huth C, Holle R. Quality of diabetes care in Germany improved from 2000 to 2007 to 2014, but improvements diminished since 2007. Evidence from the population-based KORA studies. PLoS One. 2016;11(10):e0164704.

14. Kyle UG, Genton L, Karsegard L, Slosman DO, Pichard C. Single prediction equation for bioelectrical impedance analysis in adults aged 20-94 years. Nutrition. 2001;17(3):248-53.

15. Kyle UG, Schutz Y, Dupertuis YM, Pichard C. Body composition interpretation: contributions of the fat-free mass index and the body fat mass index. Nutrition. 2003;19(7-8):597-604.

16. Janssen I, Heymsfield SB, Baumgartner RN, Ross R. Estimation of skeletal muscle mass by bioelectrical impedance analysis. J Appl Physiol (1985). 2000;89(2):465-71.

17. Levey AS, Stevens LA, Schmid CH, Zhang YL, Castro AF, 3rd, Feldman HI, et al. A new equation to estimate glomerular filtration rate. Ann Intern Med. 2009;150(9):604-12.

18. Meisinger C, Thorand B, Schneider A, Stieber J, Doring A, Lowel H. Sex differences in risk factors for incident type 2 diabetes mellitus: the MONICA Augsburg cohort study. Arch Intern Med. 2002;162(1):82-9.

19. Holz T, Thorand B, Döring A, Schneider A, Meisinger C, Koenig W. Markers of inflammation and weight change in middle-aged adults: results from the prospective MONICA/KORA S3/F3 study. Obesity. 2010;18(12):2347-53.

20. Huemer MT, Bauer A, Petrera A, Scholz M, Hauck SM, Drey M, et al. Proteomic profiling of low muscle and high fat mass: a machine learning approach in the KORA S4/FF4 study. J Cachexia Sarcopenia Muscle. 2021;12(4):1011-23.

21. Khuseyinova N, Imhof A, Trischler G, Rothenbacher D, Hutchinson WL, Pepys MB, et al. Determination of C-reactive protein: comparison of three high-sensitivity immunoassays. Clin Chem. 2003;49(10):1691-5.

22. Thorand B, Zierer A, Buyukozkan M, Krumsiek J, Bauer A, Schederecker F, et al. A panel of 6 biomarkers significantly improves the prediction of type 2 diabetes in the MONICA/KORA study population. J Clin Endocrinol Metab. 2021;106(4):e1647-e59.

23. Grimm KJ, Ram N, Estabrook R. Growth modeling: Structural equation and multilevel modeling approaches. New York: Guilford Press; 2016.

# Supplementary figures

## Figure S1 Overview of the general KORA studies


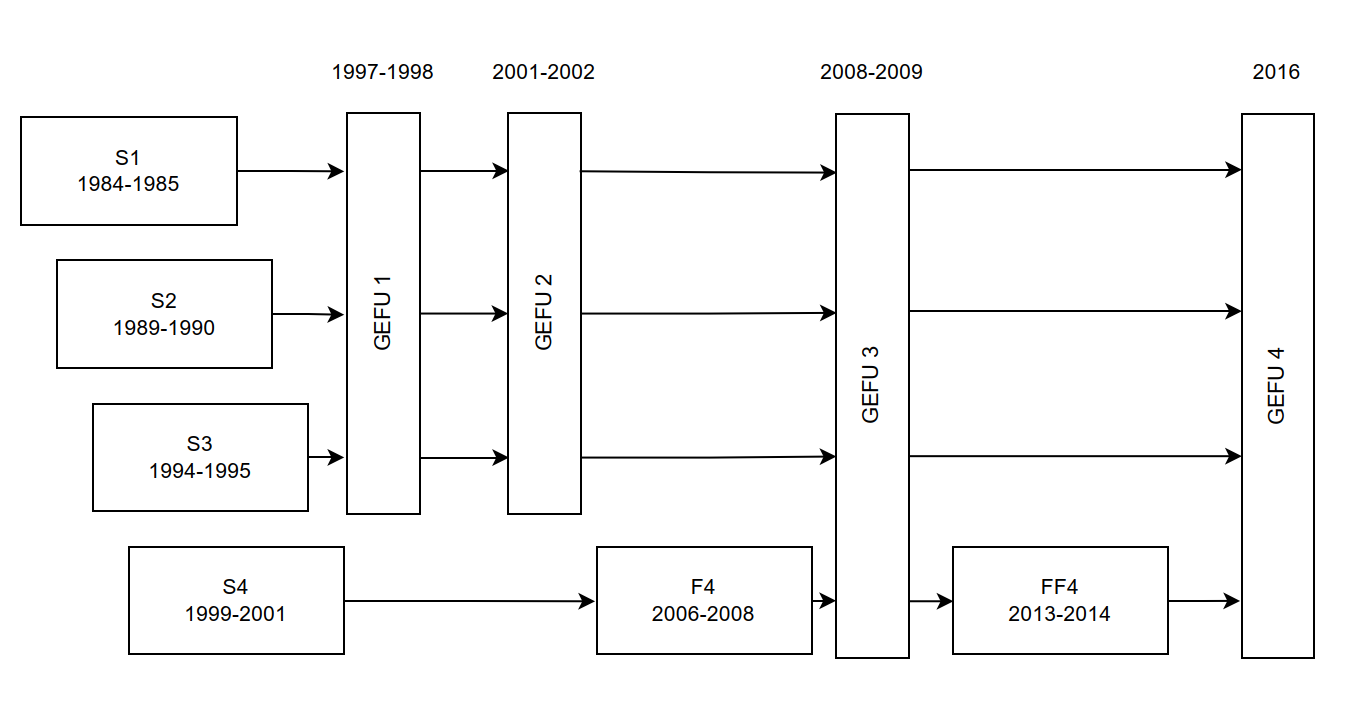


**Abbreviations:** KORA: Cooperative Health Research in the Region of Augsburg; GEFU4: self-administered health questionnaire 2016.

## Figure S2 Flowchart of the study population in the KORA S3/S4 studies


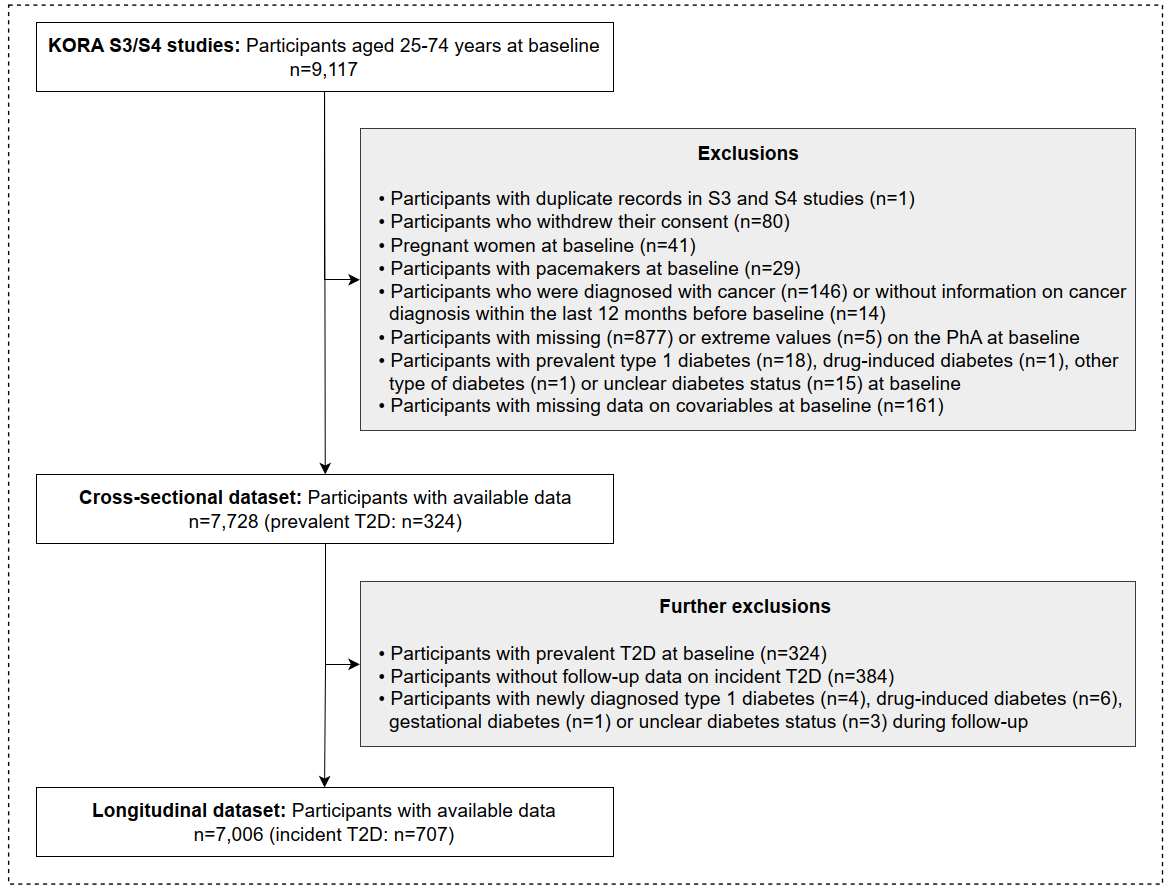


**Abbreviations**: PhA: phase angle; T2D: type 2 diabetes.

## Figure S3 Flowchart of the study population in the KORA S4/F4/FF4 studies


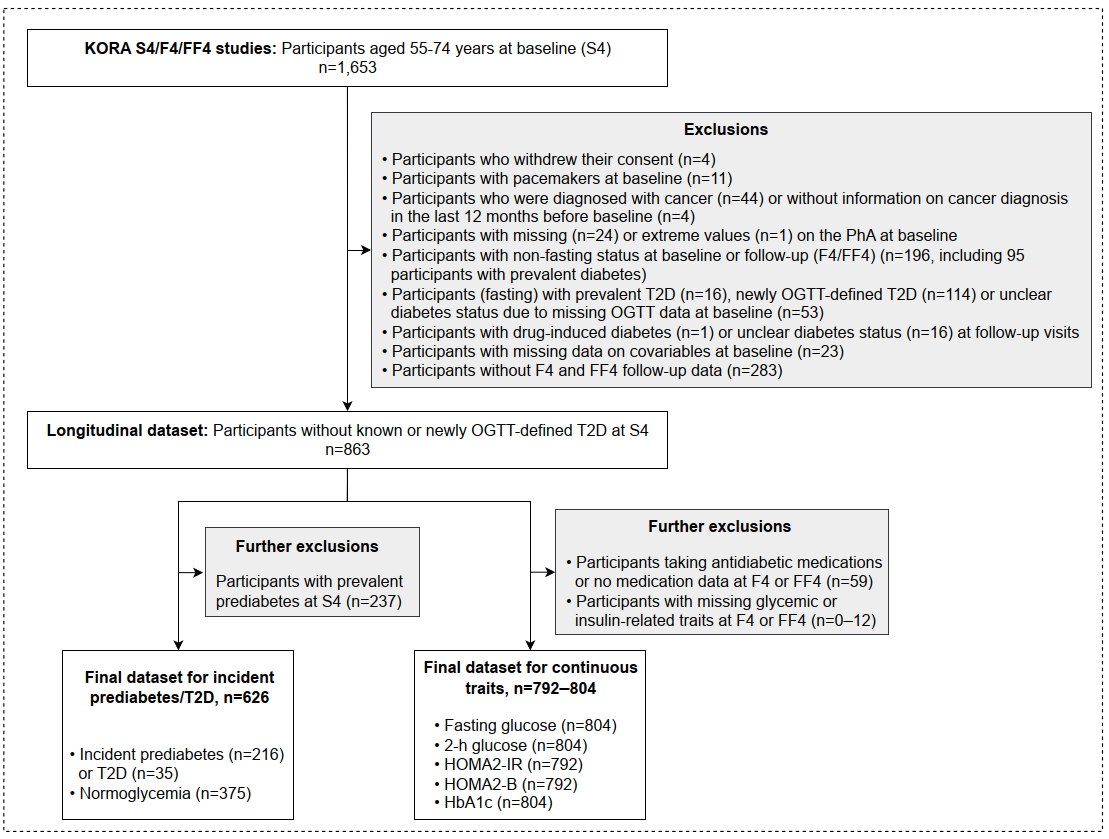


**Abbreviations**: PhA: phase angle; T2D: type 2 diabetes; OGTT: oral glucose tolerance test; 2-h glucose: 2-hour serum glucose; HOMA2-IR: updated homeostatic model assessment of insulin resistance; HOMA2-B: updated homeostatic model assessment of beta-cell function; HbA1c: glycated hemoglobin A1c.

## Figure S4 Correlation of the PhA with anthropometric and body composition parameters at baseline in the KORA S3/S4 studies


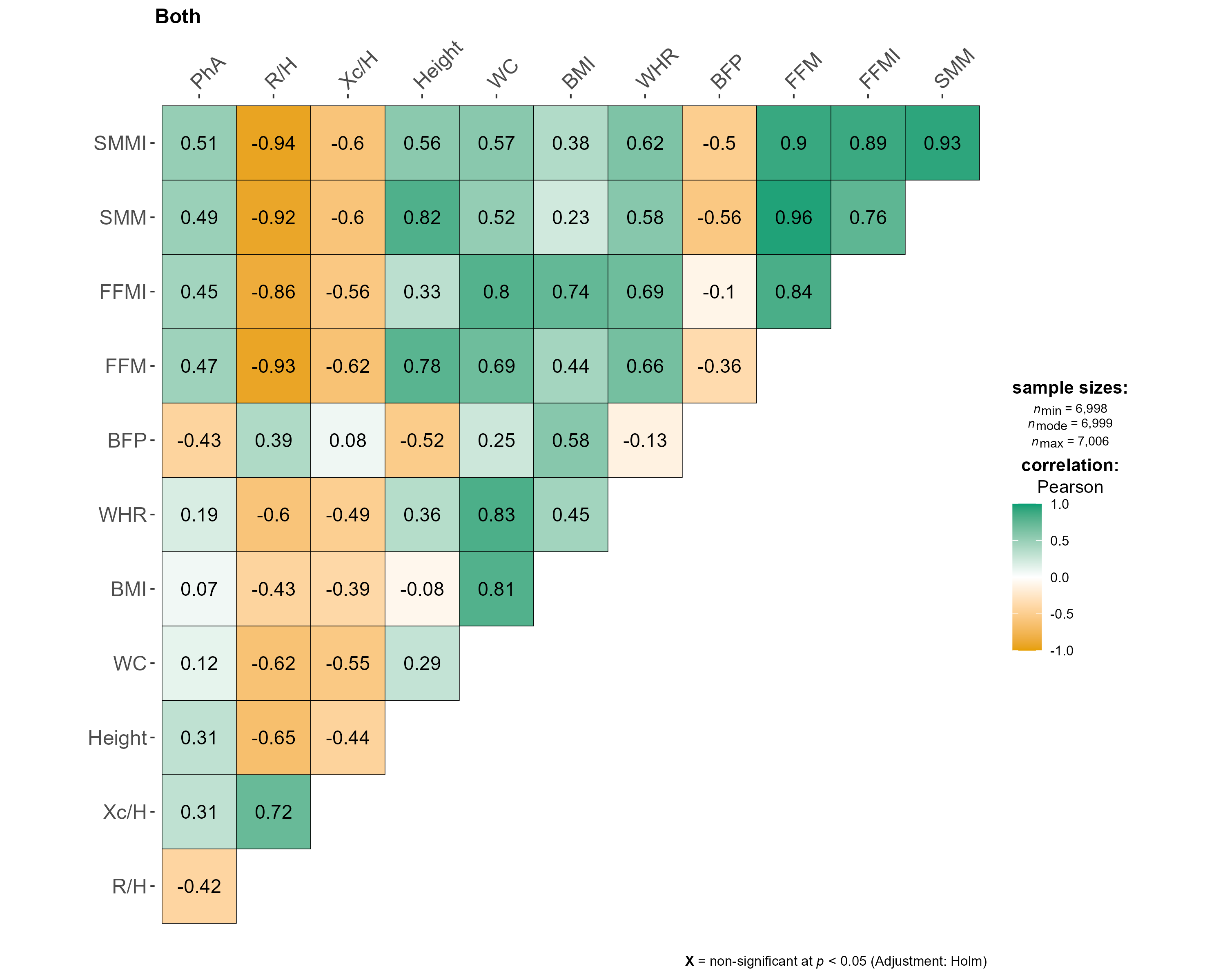


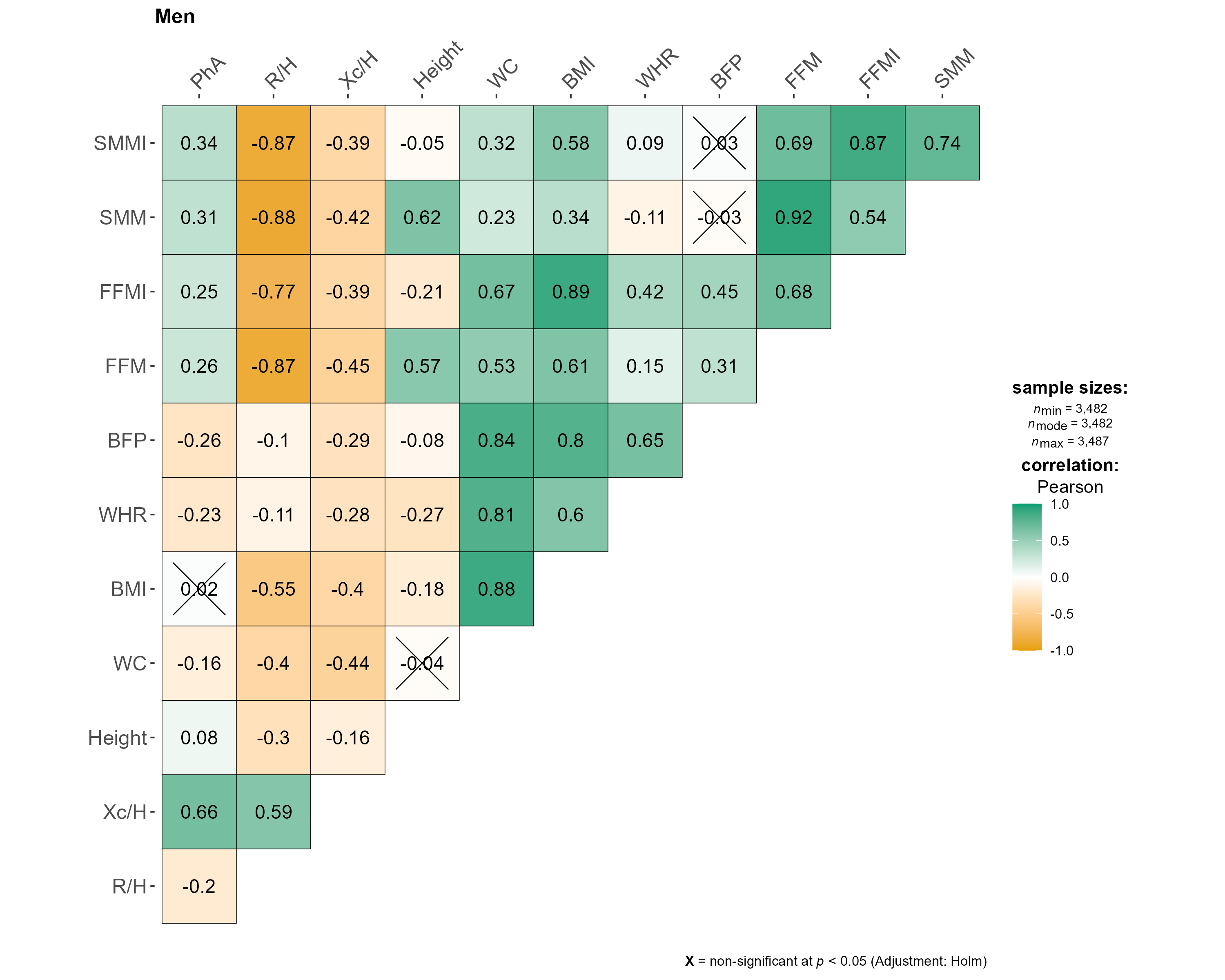


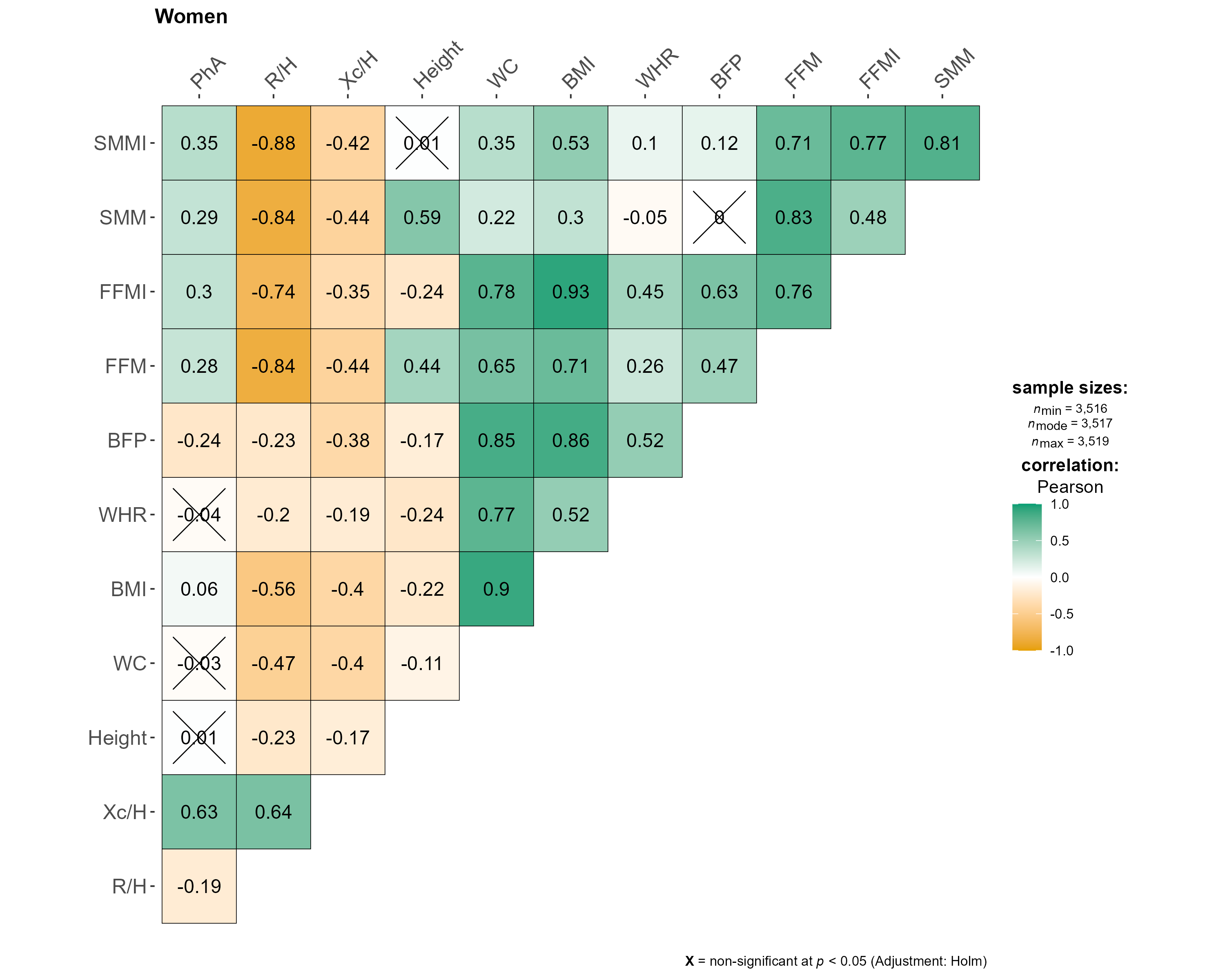


**Abbreviations:** PhA: phase angle; R/H: resistance/height; Xc/H: reactance/height; WC: waist circumference; BMI: body mass index; WHR: waist-hip ratio; BFP: body fat percentage; FFM: fat-free mass; FFMI: FFM index (by height squared); SMM: skeletal muscle mass; SMMI: SMM index (by height squared).

Pearson correlation analyses were performed separately for men and women using baseline data from the KORA S3/S4 studies, respectively. Correlations between the PhA and anthropometric, body composition, and muscle-related parameters at baseline were quantified by Pearson correlation coefficients. Green color indicates positive correlation, and yellow color indicates negative correlation.

## Figure S5 Associations of the baseline PhA with prevalent T2D and incident T2D using restricted cubic splines in the KORA S3/S4 studies

**A Cross-sectional association with prevalent T2D**


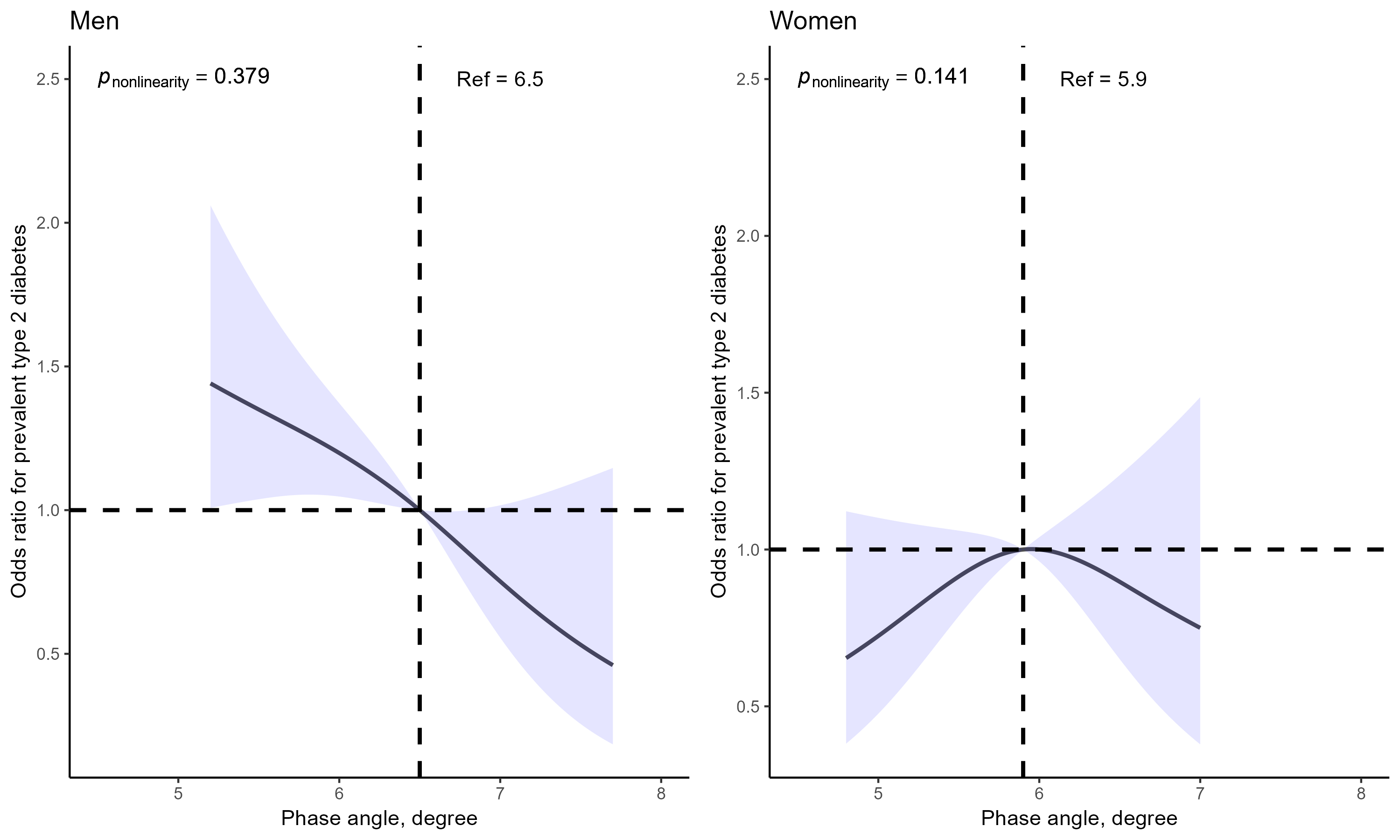


**B Longitudinal association with incident T2D**


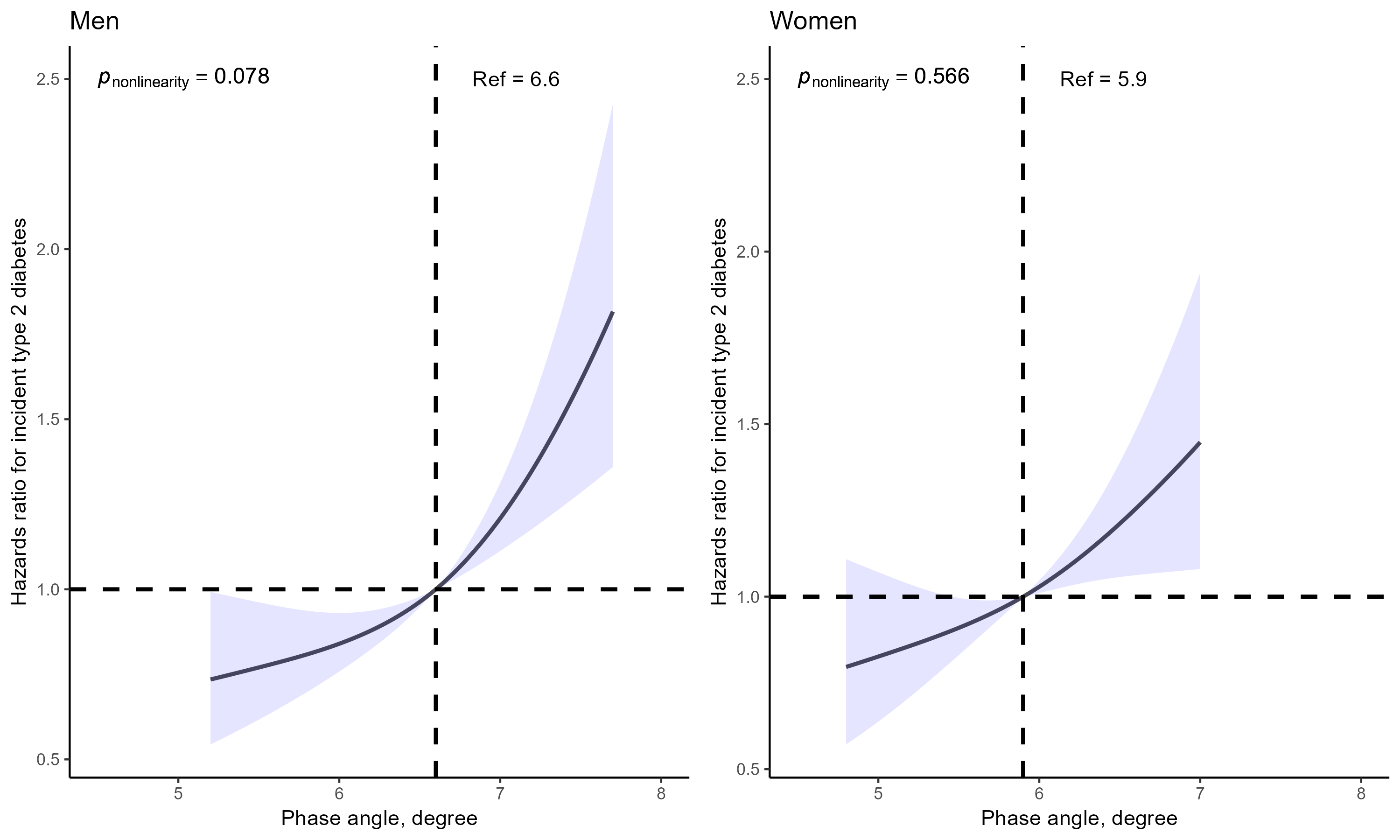


Restricted cubic splines (RCS) models were applied to assess (A) the cross-sectional association of the baseline PhA with prevalent T2D using logistic regression models (men: *n* = 3,862; women: *n* = 3,866) and (B) the longitudinal association of the baseline PhA with incident T2D using Cox proportional hazards models (men: *n* = 3,487; women: *n* = 3,519) in the KORA S3/S4 studies. The upper and lower 5% PhA values were excluded, and the median PhA values were used as reference points.

Models were adjusted for age, study, fasting status, waist circumference, smoking status, alcohol consumption, physical activity, healthy eating score, hypertension, high-density lipoprotein cholesterol, estimated glomerular ﬁltration rate, uric acid, intake of lipid-lowering medication, and parental history of diabetes.

# Supplementary tables

## Table S1 Characteristics of the study population for cross-sectional analyses in the KORA S3/S4 studies

| Characteristics | Both | |  | Men | |  | Women | |
| --- | --- | --- | --- | --- | --- | --- | --- | --- |
|  | Non-cases | Cases ^a^ |  | Non-cases | Cases ^a^ |  | Non-cases | Cases ^a^ |
| N | 7,404 | 324 |  | 3,673 | 189 |  | 3,731 | 135 |
| S4 study, *n* (%) | 3,839 (51.9) | 167 (51.5) |  | 1,879 (51.2) | 96 (50.8) |  | 1,960 (52.5) | 71 (52.6) |
| Age (years) | 48.5 (13.8) | 62.5 (8.0) ^***^ |  | 48.7 (14.0) | 63.3 (7.5) ^***^ |  | 48.4 (13.6) | 61.5 (8.6) ^***^ |
| Fasting status, yes, *n* (%) | 1,957 (26.4) | 53 (16.4) ^***^ |  | 991 (27.0) | 40 (21.2) |  | 966 (25.9) | 13 (9.6) ^***^ |
| Waist circumference (cm) | 104.0 (8.7) | 110.0 (11.8) ^***^ |  | 104.0 (6.8) | 107.6 (9.4) ^***^ |  | 104.0 (10.2) | 113.3 (13.9) ^***^ |
| Smoking status, *n* (%) |  | ^***^ |  |  | ^***^ |  |  | ^**^ |
| never | 3,142 (42.4) | 141 (43.5) |  | 1,109 (30.2) | 49 (25.9) |  | 2033 (54.5) | 92 (68.1) |
| former | 2,293 (31.0) | 132 (40.7) |  | 1,418 (38.6) | 104 (55.0) |  | 875 (23.5) | 28 (20.7) |
| current | 1,969 (26.6) | 51 (15.7) |  | 1,146 (31.2) | 36 (19.0) |  | 823 (22.1) | 15 (11.1) |
| Alcohol consumption, *n* (%) |  | ^***^ |  |  | ^**^ |  |  | ^***^ |
| no | 2,159 (29.2) | 142 (43.8) |  | 625 (17.0) | 48 (25.4) |  | 1534 (41.1) | 94 (69.6) |
| moderate | 3,696 (49.9) | 139 (42.9) |  | 2,140 (58.3) | 106 (56.1) |  | 1556 (41.7) | 33 (24.4) |
| heavy | 1,549 (20.9) | 43 (13.3) |  | 908 (24.7) | 35 (18.5) |  | 641 (17.2) | 8 (5.9) |
| Physical activity, *n* (%) |  | ^***^ |  |  | ^***^ |  |  | ^***^ |
| > 2 h/week | 1,496 (20.2) | 42 (13.0) |  | 891 (24.3) | 27 (14.3) |  | 605 (16.2) | 15 (11.1) |
| 1–2 h/week | 1,969 (26.6) | 51 (15.7) |  | 890 (24.2) | 30 (15.9) |  | 1,079 (28.9) | 21 (15.6) |
| < 1 h/week | 1,217 (16.4) | 37 (11.4) |  | 617 (16.8) | 21 (11.1) |  | 600 (16.1) | 16 (11.9) |
| none | 2,722 (36.8) | 194 (59.9) |  | 1,275 (34.7) | 111 (58.7) |  | 1,447 (38.8) | 83 (61.5) |
| Healthy eating score | 15.2 (3.6) | 16.5 (3.5) ^***^ |  | 14.5 (3.6) | 16.0 (3.5) ^***^ |  | 16.0 (3.5) | 17.3 (3.3) ^***^ |
| Hypertension, yes, *n* (%) | 2,753 (37.2) | 247 (76.2) ^***^ |  | 1,601 (43.6) | 138 (73.0) ^***^ |  | 1,152 (30.9) | 109 (80.7) ^***^ |
| HDL-C (mmol/l) | 1.5 (0.4) | 1.2 (0.4) ^***^ |  | 1.3 (0.4) | 1.1 (0.3) ^***^ |  | 1.6 (0.4) | 1.3 (0.4) ^***^ |
| eGFR (ml/min/1.73 m^2^) | 97.7 (16.8) | 87.4 (17.2) ^***^ |  | 98.2 (16.4) | 87.2 (17.0) ^***^ |  | 97.3 (17.1) | 87.7 (17.5) ^***^ |
| Uric acid (μmol/l) | 307.5 (84.4) | 337.9 (89.6) ^***^ |  | 357.1 (74.0) | 358.0 (88.2) |  | 258.6 (62.7) | 309.7 (84.1) ^***^ |
| Intake of lipid-lowering drugs, yes, *n* (%) | 293 (4.0) | 49 (15.1) ^***^ |  | 160 (4.4) | 26 (13.8) ^***^ |  | 133 (3.6) | 23 (17.0) ^***^ |
| Parental history of diabetes, *n* (%) |  | ^***^ |  |  | ^***^ |  |  | ^***^ |
| no | 4,730 (63.9) | 131 (40.4) |  | 2,357 (64.2) | 80 (42.3) |  | 2,373 (63.6) | 51 (37.8) |
| unknown | 1,166 (15.7) | 85 (26.2) |  | 636 (17.3) | 48 (25.4) |  | 530 (14.2) | 37 (27.4) |
| yes | 1,508 (20.4) | 108 (33.3) |  | 680 (18.5) | 61 (32.3) |  | 828 (22.2) | 47 (34.8) |
| PhA (°) | 6.2 (0.8) | 5.8 (0.7) ^***^ |  | 6.5 (0.8) | 5.9 (0.7) ^***^ |  | 5.9 (0.7) | 5.7 (0.7) ^***^ |
| R/H (Ω/m) | 313.2 (55.9) | 291.4 (47.4) ^***^ |  | 271.0 (31.4) | 268.2 (36.9) |  | 354.8 (41.8) | 324.0 (40.8) ^***^ |
| Xc/H (Ω/m) | 33.7 (5.8) | 29.4 (5.5) ^***^ |  | 30.9 (4.6) | 27.6 (5.0) ^***^ |  | 36.5 (5.5) | 32.1 (5.0) ^***^ |

**Abbreviations**: HDL-C: high-density lipoprotein cholesterol; eGFR: estimated glomerular filtration rate; PhA: phase angle; R/H: resistance by height; Xc/H: reactance by height.

^***^ indicates *p* < 0.001, ^**^ indicates *p* < 0.05.

^a^ Cases refer to participants with known type 2 diabetes ascertained at baseline in the KORA S3/S4 studies.

Continuous variables with normal distribution were presented as mean (standard deviation) and with skewed distribution as median [Q1, Q3]. Categorical variables were shown as n (%). T-test or Kruskal-Wallis test and Chi-square test were applied for comparison of continuous and categorical variables among cases and non-cases, respectively.

## Table S2 Pearson correlations of the PhA with age and BMI within age and BMI groups in the KORA S3/S4 studies

| Groups | N (%) | PhA (°) | *r* | *p* |
| --- | --- | --- | --- | --- |
| Age < 55 years | 4,444 (63.4) | 6.4 (0.7) | -0.17 | < 0.001 |
| Age ≥ 55 years | 2,562 (36.6) | 5.8 (0.7) | -0.36 | < 0.001 |
|  |  |  |  |  |
| BMI < 35 kg/m^2^ | 6,653 (95.1) | 6.2 (0.8) | 0.11 | < 0.001 |
| BMI ≥ 35 kg/m^2^ | 346 (4.9) | 6.2 (0.8) | -0.11 | 0.0391 |

**Abbreviations**: PhA: phase angle; BMI: body mass index.

Seven participants had missing BMI values in the final longitudinal dataset (n=6,999).

In each group, PhA values are presented as mean (standard deviation).

## Table S3 Characteristics of the study participants with continuous glycemic and insulin-related traits in the KORA S4/F4/FF4 studies

| Characteristics | Baseline (S4) | 1^st^ follow-up (F4) | 2^nd^ follow-up (FF4) |
| --- | --- | --- | --- |
| N | 804 | 792 | 477 |
| Age (years) | 63.2 (5.4) |  |  |
| Men, *n* (%) | 396 (49.3) |  |  |
| Waist circumference (cm) | 93.8 (11.1) |  |  |
| Smoking status, *n* (%) |  |  |  |
| never | 406 (50.5) |  |  |
| former | 302 (37.6) |  |  |
| current | 96 (11.9) |  |  |
| Alcohol consumption, *n* (%) |  |  |  |
| never | 194 (24.1) |  |  |
| moderate | 443 (55.1) |  |  |
| heavy | 167 (20.8) |  |  |
| Physical activity, *n* (%) |  |  |  |
| > 2 h/week | 157 (19.5) |  |  |
| 1–2 h/week | 230 (28.6) |  |  |
| < 1 h/week | 131 (16.3) |  |  |
| none | 286 (35.6) |  |  |
| Healthy eating score | 16.1 (3.5) |  |  |
| Hypertension, yes, *n* (%) | 384 (47.8) |  |  |
| HDL-C (mmol/l) | 1.5 (0.4) |  |  |
| Triglycerides (mmol/l) | 1.3 [0.9–1.7] |  |  |
| eGFR (ml/min/1.73 m^2^) | 83.1 (11.8) |  |  |
| Uric acid (μmol/l) | 328.8 (78.7) |  |  |
| Intake of lipid-lowering drugs, yes, *n* (%) | 82 (10.2) |  |  |
| Parental history of diabetes, *n* (%) |  |  |  |
| no | 475 (59.1) |  |  |
| unknown | 149 (18.5) |  |  |
| yes | 180 (22.4) |  |  |
| Fasting glucose (mmol/l) | 5.4 [5.1–5.8] | 5.4 [5.1–5.8] | 5.6 [5.2–6.0] |
| 2-h glucose (mmol/l) | 6.1 [5.1–7.1] | 6.6 [5.5–8.1] | 7.0 [5.7–8.6] |
| HOMA2-IR | 1.1 [0.8–1.5] | 1.1 [0.8–1.6] | 1.2 [0.8–1.6] |
| HOMA2-B | 84.9 [68.8–104.8] | 87.0 [69.5–105.9] | 82.2 [66.2–103.2] |
| HbA1c (%) | 5.6 [5.4–5.8] | 5.6 [5.4–5.8] | 5.5 [5.3–5.8] |
| HbA1c (mmol/mol) | 38.0 [36.0–40.0] | 38.0 [36.0–40.0] | 37.0 [35.0–40.0] |
| PhA (°) | 5.8 (0.7) |  |  |
| R/H (Ω/m) | 316.7 (54.4) |  |  |
| Xc/H (Ω/m) | 32.0 (5.7) |  |  |

**Abbreviations**: HDL-C: high-density lipoprotein cholesterol; eGFR: estimated glomerular ﬁltration rate; 2-h glucose: 2-hour serum glucose; HOMA2-IR: updated homeostatic model assessment of insulin resistance; HOMA2-B: updated homeostatic model assessment of beta-cell function; HbA1c: glycated hemoglobin A1c; PhA: phase angle; R/H: resistance by height; Xc/H: reactance by height.

Continuous variables with normal distribution are presented as mean (standard deviation) and with skewed distribution as median [Q1–Q3]. Categorical variables are shown as n (%). The five continuous traits were described in their original scale.

## Table S4 Stratified analyses of the longitudinal association of the baseline PhA with incident T2D in the KORA S3/S4 studies

|  | Incident T2D | | |
| --- | --- | --- | --- |
|  | Group 1 | Group 2 | *p* _interaction_ |
| Age groups |  |  |  |
|  | Age < 55 years | Age ≥ 55 years |  |
| N | 4,444 | 2,562 |  |
| Cases/person-years | 297/71,467 | 410/33,409 |  |
| HR [95% CI] |  |  |  |
| Model 1 | 1.69 [1.40–2.04] ^***^ | 1.36 [1.16–1.58] ^***^ | 0.975 |
| Model 2 | 1.59 [1.33–1.91] ^***^ | 1.31 [1.12–1.53] ^**^ | 0.920 |
| Model 3 | 1.50 [1.24–1.82] ^***^ | 1.24 [1.05–1.45] ^**^ | 0.735 |
| BMI groups |  |  |  |
|  | BMI < 35 kg/m^2^ | BMI ≥ 35 kg/m^2^ |  |
| N | 6,653 | 346 |  |
| Cases/person-years | 592/100,748 | 112/4,058 |  |
| HR [95% CI] |  |  |  |
| Model 1 | 1.56 [1.37–1.77] ^***^ | 1.16 [0.84–1.60] | 0.981 |
| Model 2 | 1.45 [1.27–1.65] ^***^ | 1.25 [0.89–1.75] | 0.690 |
| Model 3 | 1.38 [1.21–1.58] ^***^ | 1.21 [0.86–1.72] | 0.914 |

**Abbreviations**: PhA: phase angle; T2D: type 2 diabetes; HR: hazard ratio; CI: confidence interval; BMI: body mass index.

^***^ indicates *p* < 0.001, ^**^ indicates *p* < 0.05.

Incident T2D (S3/S4): known T2D ascertained during follow-up until 2016.

The HR and 95% CI are per 1-degree increase of the baseline PhA.

Model 1: adjusted for age, sex, study, and fasting status.

Model 2: adjusted for variables in model 1 plus waist circumference, smoking status, alcohol consumption, physical activity, and healthy eating score.

Model 3: adjusted for variables in model 2 plus hypertension, high-density lipoprotein cholesterol, estimated glomerular ﬁltration rate, uric acid, intake of lipid-lowering medication, and parental history of diabetes.

## Table S5 Sensitivity analyses of the longitudinal association of the baseline PhA with incident T2D in the KORA S3/S4 studies or incident prediabetes/T2D in the KORA S4/F4/FF4 studies

|  | Incident T2D | | Incident prediabetes/T2D | |
| --- | --- | --- | --- | --- |
|  | Cases/N | HR [95% CI] | Cases/N | HR [95% CI] |
| Exclusion of 103 participants with follow-up time < 2 years | 640/6,903 | 1.38 [1.21–1.56] ^***^ |  |  |
| Accounting for death as a competing risk | 704/6,999 | 1.44 [1.27–1.63] ^***^ |  |  |
| Alternative adjustment |  |  |  |  |
| + WHR ^a^ | 707/7,005 | 1.33 [1.18–1.49] ^***^ | 251/626 | 1.32 [1.06-1.64] ^**^ |
| + BMI ^a^ | 704/6,999 | 1.30 [1.15–1.47] ^***^ | 251/626 | 1.31 [1.05–1.65] ^**^ |
| + BFP ^a^ | 704/6,999 | 1.62 [1.43–1.84] ^***^ | 251/626 | 1.40 [1.10–1.77] ^**^ |
| + FFM ^a^ | 704/6,999 | 1.33 [1.18–1.50] ^***^ | 251/626 | 1.33 [1.07–1.65] ^**^ |
| + FFMI ^a^ | 704/6,999 | 1.13 [1.00–1.28] | 251/626 | 1.25 [1.00–1.56] |
| + SMM ^a^ | 705/7,004 | 1.40 [1.24–1.58] ^***^ | 251/626 | 1.33 [1.07–1.66] ^**^ |
| + SMMI ^a^ | 705/7,004 | 1.27 [1.12–1.44] ^***^ | 251/626 | 1.31 [1.06–1.63] ^**^ |
| + triglycerides ^b^ | 537/6,444 | 1.40 [1.22–1.61] ^***^ |  |  |
| + albumin | 699/6,920 | 1.39 [1.24–1.57] ^***^ | 250/622 | 1.34 [1.07–1.70] ^**^ |
| + hs-CRP ^b^ | 699/6,909 | 1.39 [1.23–1.57] ^***^ | 250/622 | 1.34 [1.06–1.69] ^**^ |
| + NT-proBNP ^b^ | 233/4,718 | 1.44 [1.15–1.79] ^**^ | 300/490 | 1.45 [1.10–1.92] ^**^ |
| + intake of diuretics | 707/7,006 | 1.37 [1.21–1.54] ^***^ | 251/626 | 1.33 [1.07–1.67] ^**^ |

**Abbreviations**: PhA: phase angle; T2D: type 2 diabetes; HR: hazard ratio; CI: confidence interval; WHR: waist-hip ratio; BMI: body mass index; BFP: body fat percentage; FFM: fat-free mass; FFMI: FFM index (by height squared); SMM: skeletal muscle mass; SMMI: SMM index (by height squared); hs-CRP: high-sensitivity C-reactive protein; NT-proBNP: N-terminal pro-B-type natriuretic peptide.

^***^ indicates *p* < 0.001, ^**^ indicates *p* < 0.05.

Incident T2D (S3/S4): known T2D ascertained during follow-up until 2016.

Incident prediabetes/T2D (S4/F4/FF4): OGTT-defined prediabetes or OGTT-defined T2D identified at follow-up visits (F4 or FF4) during follow-up until the end of FF4 or known T2D ascertained during follow-up until the end of FF4.

The HR and 95% CI are per 1-degree increase of the baseline PhA.

^a^ Waist circumference was removed due to collinearity.

^b^ Variables were transformed using the natural logarithm due to their skewed distribution.

Models were adjusted for age, sex, study, fasting status, waist circumference, smoking status, alcohol consumption, physical activity, healthy eating score, hypertension, high-density lipoprotein cholesterol, estimated glomerular ﬁltration rate, uric acid, intake of lipid-lowering medication, and parental history of diabetes (Model 3).

## Table S6 Associations of the baseline PhA with glycemic and insulin-related traits among participants without diabetes at baseline in the KORA S4/F4/FF4 studies

| Continuous traits | N | Cross-sectional associations | |  | Longitudinal associations | |
| --- | --- | --- | --- | --- | --- | --- |
|  |  | Beta [95% CI] | *p* |  | Beta [95% CI] | *p* |
| Fasting glucose | 804 |  |  |  |  |  |
| Model 1 |  | 0.0193 [0.0086–0.0300] | <0.001 |  | 0.0025 [-0.0053–0.0104] | 0.527 |
| Model 2 |  | 0.0141 [0.0037–0.0246] | 0.008 |  | 0.0025 [-0.0053–0.0104] | 0.526 |
| Model 3 |  | 0.0117 [0.0013–0.0222] | 0.028 |  | 0.0025 [-0.0053–0.0104] | 0.525 |
| 2-h glucose | 804 |  |  |  |  |  |
| Model 1 |  | 0.0050 [-0.0244–0.0345] | 0.738 |  | 0.0448 [0.0225–0.0672] | <0.001 |
| Model 2 |  | -0.0042 [-0.0328–0.0244] | 0.773 |  | 0.0450 [0.0227–0.0674] | <0.001 |
| Model 3 |  | -0.0102 [-0.0383–0.0179] | 0.476 |  | 0.0449 [0.0225–0.0672] | <0.001 |
| HOMA2-IR | 792 |  |  |  |  |  |
| Model 1 |  | 0.1250 [0.0690–0.1810] | <0.001 |  | -0.0119 [-0.0460–0.0223] | 0.496 |
| Model 2 |  | 0.0900 [0.0421–0.1380] | <0.001 |  | -0.0109 [-0.0450–0.0231] | 0.529 |
| Model 3 |  | 0.0702 [0.0232–0.1172] | 0.004 |  | -0.0111 [-0.0451–0.0230] | 0.524 |
| HOMA2-B | 792 |  |  |  |  |  |
| Model 1 |  | 0.0456 [0.0082–0.0829] | 0.017 |  | -0.0129 [-0.0361–0.0103] | 0.277 |
| Model 2 |  | 0.0326 [-0.0014–0.0666] | 0.061 |  | -0.0120 [-0.0352–0.0111] | 0.309 |
| Model 3 |  | 0.0238 [-0.0100–0.0576] | 0.168 |  | -0.0121 [-0.0353–0.0110] | 0.305 |
| HbA1c | 804 |  |  |  |  |  |
| Model 1 |  | 0.0053 [-0.0012–0.0118] | 0.112 |  | 0.0024 [-0.0030–0.0078] | 0.386 |
| Model 2 |  | 0.0047 [-0.0018–0.0113] | 0.158 |  | 0.0024 [-0.0030–0.0078] | 0.384 |
| Model 3 |  | 0.0024 [-0.0041–0.0089] | 0.472 |  | 0.0024 [-0.0030–0.0078] | 0.384 |

**Abbreviations**: PhA: phase angle; CI: confidence interval; 2-h glucose:2-hour serum glucose; HOMA2-IR: updated homeostatic model assessment of insulin resistance; HOMA2-B: updated homeostatic model assessment of beta-cell function; HbA1c: glycated hemoglobin A1c.

The beta and 95% CI are per 1-degree increase of the baseline PhA corresponding to log_e_-transformed continuous traits. Longitudinal (within-participant) effects refer to the association between the baseline PhA and changes in the five continuous traits over a 10-year period; cross-sectional (between-participant) effects refer to the association between the PhA and variations of the five continuous traits at baseline.

Model 1: adjusted for age and sex.

Model 2: adjusted for variables in model 1 plus waist circumference, smoking status, alcohol consumption, physical activity, and healthy eating score.

Model 3: adjusted for variables in model 2 plus hypertension, high-density lipoprotein cholesterol, log_e_-transformed triglycerides, estimated glomerular ﬁltration rate, uric acid, intake of lipid-lowering medication, and parental history of diabetes.
